# A novel approach to oxoisoaporphine alkaloids via regioselective metalation of alkoxy isoquinolines

**DOI:** 10.3762/bjoc.13.156

**Published:** 2017-08-08

**Authors:** Benedikt C Melzer, Franz Bracher

**Affiliations:** 1 Department für Pharmazie - Zentrum für Pharmaforschung, Ludwig-Maximilians Universität München, Butenandtstr. 5-13, D-81377 Munich, Germany

**Keywords:** directed *ortho*/remote metalation, Eaton’s reagent, isoquinolines, Suzuki cross-coupling

## Abstract

Oxoisoaporphine alkaloids are conveniently prepared via direct ring metalation of alkoxy-substituted isoquinolines at C-1, followed by reaction with iodine. Subsequent Suzuki cross-coupling of the resulting 1-iodoisoquinolines to methyl 2-(isoquinolin-1-yl)benzoates and intramolecular acylation of the corresponding carboxylic acids with Eaton’s reagent afforded five alkaloids of the oxoisoaporphine type. The yield of the cyclization step strongly depends on the electrophilic properties of ring B. An alternative cyclization protocol via directed remote metalation of ester and amide intermediates was investigated thoroughly, but found to be not feasible. Two of the alkaloids showed strong cytotoxicity against the HL-60 tumor cell line.

## Introduction

Benzylisoquinoline alkaloids represent a very large group of plant secondary metabolites, which includes about 2,500 known structures. Besides simple benzylisoquinolines, more complex tetracyclic ring systems like aporphines, protoberberines, cularines, pavines, as well as pentacyclic morphinane-type alkaloids belong to this class. Biosynthetically, all of these chemotypes are derived from the 1-benzyltetrahydroisoquinoline (*S*)-norcoclaurine. The structures, biosynthesis and pharmacology of benzylisoquinoline alkaloids have been reviewed recently by Hagel and Facchini [1].

**Figure 1:**
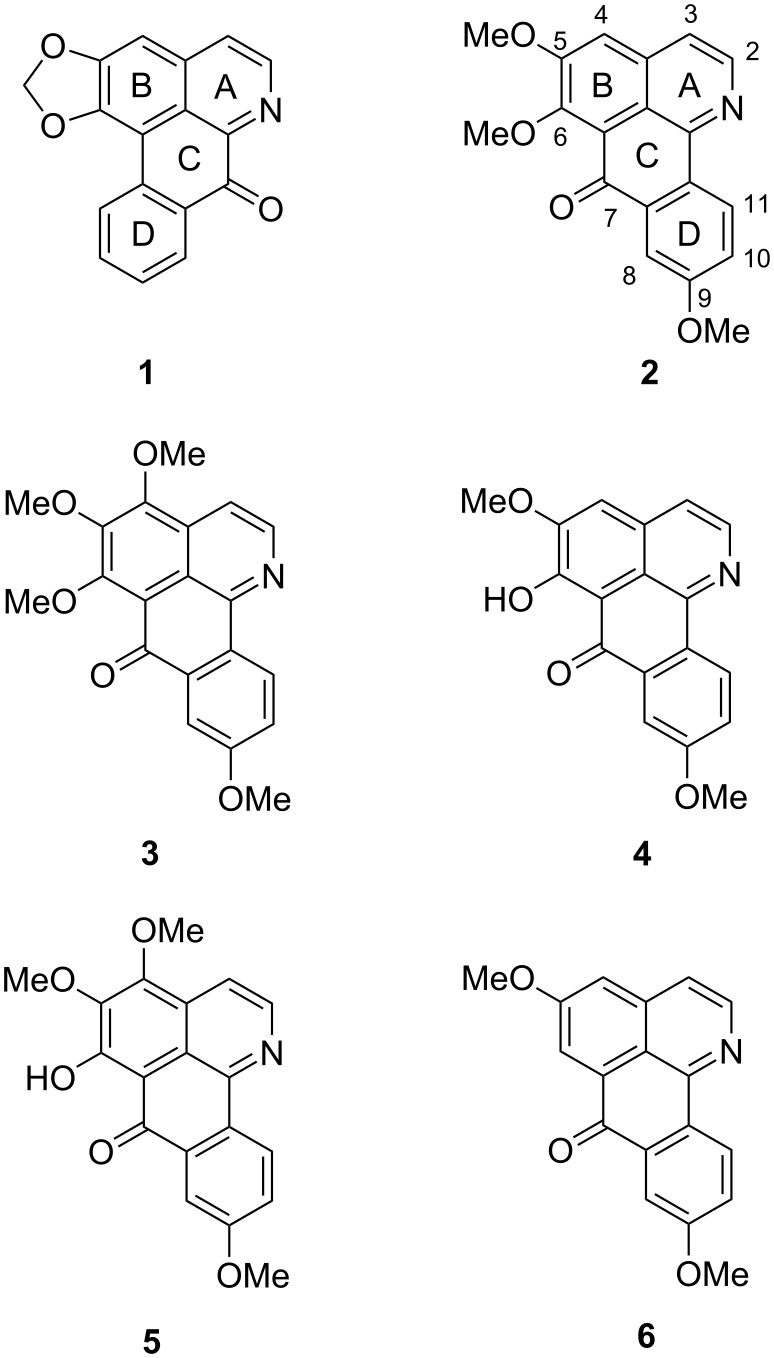
Prominent oxoaporphine and oxoisoaporphine alkaloids: liriodenine (**1**), menisporphine (**2**), dauriporphine (**3**), 6-*O*-demethylmenisporphine (**4**), dauriporphinoline (**5**), and bianfugecine (**6**).

The subgroup of aporphinoid alkaloids [2] consists of the aporphines and oxoaporphines (e.g., liriodenine (**1**)), as well as several truncated chemotypes like aristolactams and azafluoranthenes, and is of considerable pharmacological interest due to the significant cytotoxicity of numerous representatives [3]. A unique subclass of the aporphinoid alkaloids are the oxoisoaporphines (7*H*-dibenzo[*de*,*h*]quinolin-7-ones, e.g., menisporphine, (**2**)), which at the first glance appear to be not derived from 1-benzyltetrahydroisoquinoline intermediates, albeit a hypothesis of Kunitomo postulates a biosynthesis from a benzylisoquinoline precursor involving a rearrangement (Figure 1) [4].

Menisporphine (**2**), first isolated from *Menispermum dauricum* DC [4], shows antiangiogenic activity. Some, partly synthetic, oxoisoaporphine-like analogues were found to have strong DNA binding affinity and therefore high cytotoxicity [5] as well as antiplasmodial activity [6]. Besides menisporphine (**2**), the related oxoisoaporphine alkaloids dauriporphine (**3**), 6-*O*-demethylmenisporphine (**4**), dauriporphinoline (**5**) and bianfugecine (**6**) were isolated from *Menispermum dauricum* DC, among a few other plants [1,7–8].

A number of approaches to the oxoaporphine ring system has been published, typically involving the synthesis of 1-benzyl/1-benzoylisoquinoline intermediates, followed by cyclization under intramolecular biaryl synthesis, utilizing either photochemical [9–13], radical [14] or Pd-catalyzed [15–16] reactions.

**Scheme 1:**
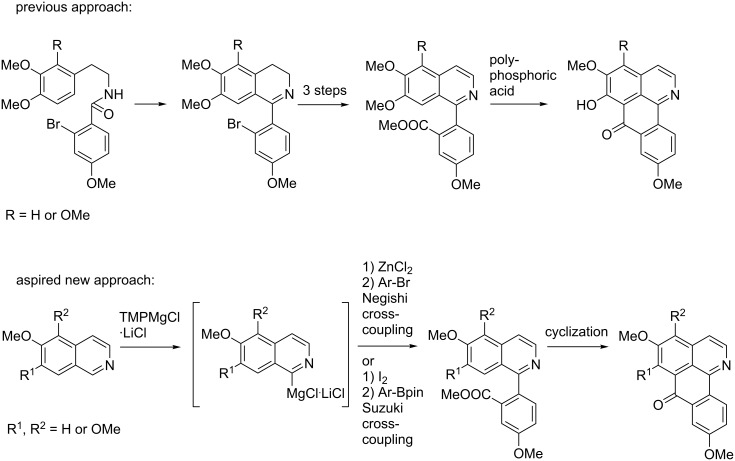
Previously reported [7,17] and new approach to oxoisoaporphine alkaloids.

In contrast, only minute work has been published on the synthesis of oxoisoaporphines. The approaches are mainly based on Kunitomo’s total syntheses of menisporphine (**2**) [7] and dauriporphine (**3**) [17], which start with the synthesis of 1-(2-bromoaryl)isoquinolines using Bischler–Napieralski chemistry, followed by tedious replacement of the bromine substituent by cyanide (for modern variants, see ref. [18–19]), and subsequent conversion to a carboxylate and Friedel–Crafts-type cyclization using polyphosphoric acid (Scheme 1). In the course of this cyclization, the methoxy group at C-6 is typically hydrolized to the phenol, subsequent *O*-methylation gives the 6-methoxy derivatives menisporphine (**2**) and dauriporphine (**3**). Recently, Zhang et al. [20] described an alternative approach starting from an isoquinoline bearing an ester group at C-8. In a photoredox-catalyzed direct C–H arylation a 4-methoxyphenyl residue from a methoxyphenyldiazonium salt was introduced at C-1, and after ester hydrolysis intramolecular Friedel–Crafts acylation afforded menisporphine (**2**).

In continuation of our recent work on the synthesis of polycyclic aromatic alkaloids like pyridoacridine, benzylisoquinoline-type and oxoaporphine alkaloids using direct ring metalations of heterocycles as vital step [13,21] we intended to develop a novel and more flexible access to oxoisoaporphine alkaloids. Again we were inspired by Knochel’s reports on the direct metalation of isoquinoline [22] and 6,7-dimethoxyisoquinoline [23] as well as by our own results for the metalation of various alkoxyisoquinolines [13] at C-1 with the Knochel–Hauser base TMPMgCl·LiCl. Transmetalation of the intermediate organomagnesium species with ZnCl_2_ should lead to at C-1 zincated isoquinolines, which could undergo Negishi cross-coupling reactions with appropriately substituted methyl 2-bromobenzoates to give methyl 2-(isoquinolin-1-yl)benzoates. These would again open an access to oxoisoaporphine alkaloids via intramolecular acylation following Kunitomo’s strategy. Main advantage of this approach should be that the laborious introduction of the carboxy residue at a late stage of the total synthesis is circumvented. Alternatively, quenching of the 1-magnesiated alkoxyisoquinolines with iodine should lead to 1-iodoisoquinolines, which were expected to be versatile substrates for Suzuki cross-coupling reactions with appropriate phenylboronic acids to gain methyl 2-(isoquinolin-1-yl)benzoates as the central intermediates (Scheme 1).

## Results and Discussion

As we reported previously, metalation of 6,7-dimethoxyisoquinoline (**7a**) and 5,6,7-trimethoxyisoquinoline (**7b**) with 1.5 equivalents of the Knochel–Hauser base (TMPMgCl∙LiCl) exclusively occurs at C-1. Trapping with appropriate benzaldehydes opened an access to benzylisoquinoline and oxoaporphine-type alkaloids [13]. Here we describe the extension of this approach to the synthesis of oxoisoaporphines.

**Scheme 2:**
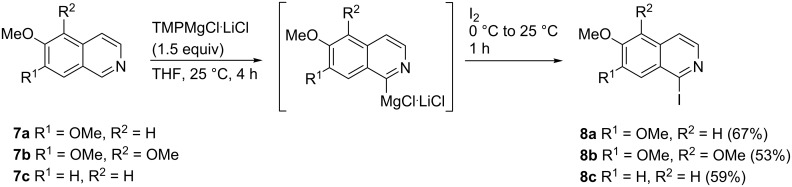
Synthesis of iodinated isoquinolines **8a–c** from alkoxy-substituted isoquinolines **7a–c**.

By using our modification (1.5 equivalents of TMPMgCl∙LiCl over 4 h at room temperature) [13] of Knochel’s [23] protocol for the metalation of isoquinolines, the alkoxy-substituted isoquinolines **7a**,**b** were metalated at C-1, as shown before, and this method could conveniently be extended to 6-methoxyisoquinoline (**7c**). Unfortunately, transmetalation with ZnCl_2_ at 0 °C followed by palladium-catalyzed (5 mol % Pd(dba)_2_/ 10 mol % P(2-furyl)_3_ or 1 mol % Pd_2_(dba)_3_/2 mol % RuPhos) Negishi cross-coupling reaction with methyl 2-bromo-5-methoxybenzoate did not lead to the expected methyl 2-(isoquinolin-1-yl)benzoates **10** after up to 72 h at room temperature or 60 °C [22]. Starting materials were recovered almost quantitatively. However, quenching with iodine gave the 1-iodoisoquinolines **8a** [23], **8b**, and **8c** in good yields (53–67%, Scheme 2).

**Scheme 3:**
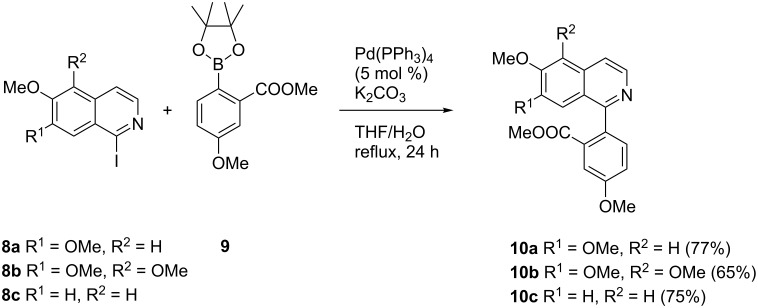
Synthesis of methyl 2-(isoquinolin-1-yl)benzoates **10a–c** from 1-iodoisoquinolines **8a–c**.

For the introduction of ring D of the oxoisoaporphine alkaloids one common building block, (4-methoxy-2-(methoxycarbonyl)phenyl)boronic acid pinacol ester (**9**) [24], could be applied, since the alkaloids of interest all bear the methoxy group at C-9. Suzuki cross-coupling reaction of the iodinated isoquinolines **8a**–**c** with this boronate under Pd(PPh_3_)_4_ catalysis gave the desired 1-arylisoquinolines **10a–c** in moderate to good isolated yields (65–77%, Scheme 3). Up to 20% of the starting materials **8a**–**c** were typically recovered, but all attempts to achieve complete conversion (longer reaction times, other catalysts and bases) were in vain.

**Scheme 4:**
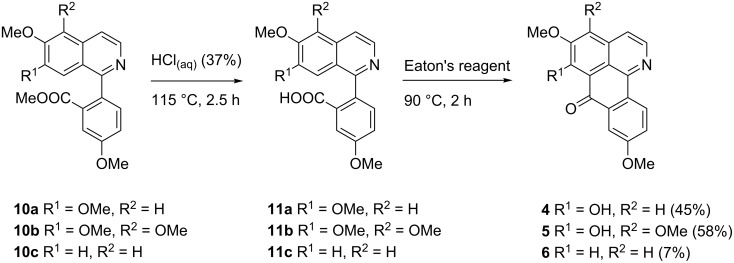
Synthesis of the alkaloids 6-*O*-demethylmenisporphine (**4**), dauriporphinoline (**5**), and bianfugecine (**6**).

The esters **10a–c** are valuable intermediates for the further conversion to the target oxoisoaporphines, and in previous protocols [7,17] the esters were typically converted into the corresponding carboxylic acids, and then cyclized using polyphosphoric acid. Previously we found that esters can directly be subjected to this type of intramolecular Friedel–Crafts-type acylations, and trifluoromethanesulfonic acid [25] is superior to polyphosphoric acid [26–27] for the synthesis of polycyclic ketones. Unfortunately, direct cyclization of esters **10a–c** using this reagent failed completely, despite numerous variations of the reaction conditions. As an alternative reagent for the acylation of arenes with esters Eaton’s reagent (phosphorus pentoxide, 7.7 wt % in methanesulfonic acid) has been described in the literature [28–29]. Eaton’s reagent has advantages over polyphosphoric acid as it is an easy to handle, non-viscous and freely measurable liquid, and therefore more suitable for small-scale synthesis. However, attempted direct cyclization of ester **10a** with Eaton’s reagent to menisporphine (**2**) failed. Consequently, we had to come back to Kunitomo’s approach [7,17] via carboxylate intermediates. Carboxylic acid **11a** was conveniently obtained by hydrolysis of the methyl ester **10a** with concentrated hydrochloric acid. The crude carboxylic acid **11a** underwent cyclization to 6-*O*-demethylmenisporphine (**4**) with Eaton’s reagent in 45% yield over both steps (Scheme 4). In accordance with Kunitomo’s observations [7,17], we also observed demethylation of the methoxy group at C-6. Dauriporphinoline (**5**) was synthesized in 58% yield using the same protocol starting from the methyl ester **10b**, once again under hydrolysis of the 6-methoxy group.

In order to explore the scope of this new cyclization methodology, we further investigated the synthesis of the alkaloid bianfugecine (**6**, Scheme 4), which contains only one methoxy group at ring B. Until now, only one partial synthesis of this alkaloid is described in the literature. Kunitomo et al. [30] obtained alkaloid **6** by hydrogenolytic demethoxylation of alkaloid menisporphine (**2**) with H_2_/PtO_2_ in acetic acid. Unfortunately, upon cyclization of the carboxylic acid **11c**, obtained by acidic hydrolysis of ester **10c**, with Eaton’s reagent alkaloid **6** could be isolated in only very poor yield (7%). Other established cyclization methods starting from methyl ester **10c** were tested in order to increase the yield. As described above, attempted direct intramolecular Friedel–Crafts-type cyclization of **10c** catalyzed by trifluoromethanesulfonic acid [25] failed completely. Also, generation of an acid chloride from **11c** with thionyl chloride, followed by the reaction with AlCl_3_ did not even give traces of bianfugecine (**6**).

Taken all of these results together, intramolecular Friedel–Crafts-type cyclization aimed at the construction of the oxoisoaporphine scaffold is a very challenging conversion, and the electronic properties of the ring to be attacked (ring B) strongly determine the outcome of this reaction.

**Scheme 5:**
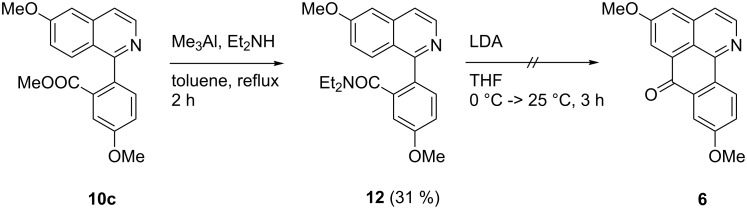
Attempted synthesis of bianfugecine (**6**) via directed remote metalation and subsequent trapping of the carboxamide group.

Inspired by a report from the Snieckus group [31] on the application of the directed remote metalation (DreM) methodology for generation of carbanionic Friedel–Crafts equivalents (generating acridones from diarylamine carboxamides), we converted methyl ester **10c** into the corresponding diethyl amide **12**. For this purpose, ester **10c** was reacted in a Weinreb amidation [32] with a mixture of trimethylaluminium and diethylamine to give the amide **12** in 31% yield (Scheme 5). The diethyl amide moiety was designated to promote a directed remote metalation by lithium diisopropylamide (LDA) at C-8 of the isoquinoline ring, which should be followed by an intramolecular trapping of the amide group to give the oxoisoaporphine bianfugecine (**6**, Scheme 5). However, only starting material **12** was recovered from this reaction.

**Scheme 6:**
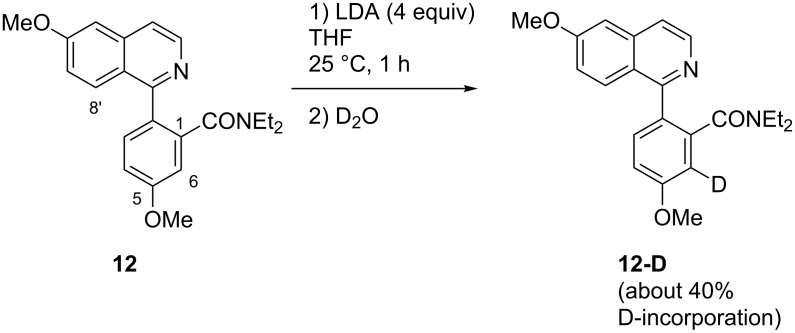
Outcome of a D_2_O quenching experiment after metalation of amide **12**.

A D_2_O quenching experiment after the metalation period (4 equiv LDA, 1 h, 25 °C) was performed in order to identify the position(s) of ring metalation of **12**. In recovered (about 80%) educt, deuterium incorporation (about 40%; calculated from integrals of the ^1^H NMR spectrum) was observed exclusively at C-6 of the benzamide moiety (Scheme 6). It is known for decades [33] that cooperative effects of directing groups lead to directed *ortho*-metalation (DoM) at their common *ortho*-position (C-6 in this case, located *ortho* to both the amide and the methoxy group). Nevertheless, under reversible metalation conditions, typically existing when amide bases like LDA are employed, kinetically controlled DoM can be followed by an equilibration to give a thermodynamically controlled DreM product [34]. This has been demonstrated by Tilly et al. [35] in the synthesis of fluorenones by treatment of *N*,*N*-dialkyl biphenyl-2-carboxamides with LDA. In our case, however, initial metalation at C-6 of the benzamide moiety was not followed by an equilibration giving the desired 8’-metalated intermediate (which in turn should be trapped by the amide group to give the tetracyclic ketone **6**). Probably, the DreM at C-8’ is prevented, since the amide moiety forms a chelate with a lithium ion and N-2 of the isoquinoline moiety, thus is kept away from C-8’. In contrast, we found in previous investigations on the synthesis of the pyridoacridone alkaloid demethyldeoxyamphimendine [21], that an ester group located at a comparable position (suitable for forming a chelate in cooperation with a pyridine nitrogen), can very well promote a directed remote metalation.

**Scheme 7:**
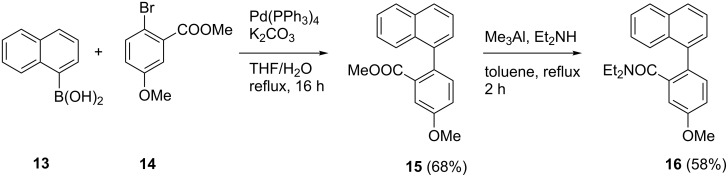
Synthesis of 1-arylnaphthalene analogues **15** and **16**.

To test our hypothesis, we performed two more D_2_O quenching experiments with naphthalene analogues of esters **10** and amide **12**. The methyl ester analogue **15** was obtained by Suzuki cross-coupling of naphthalene-1-boronic acid (**13**) and methyl 2-bromo-5-methoxybenzoate (**14**) in 68% yield. Methyl ester **15** was converted into the corresponding diethyl amide **16** in 58% yield by Weinreb amidation following the protocol described above for the synthesis of **12** (Scheme 7).

**Scheme 8:**
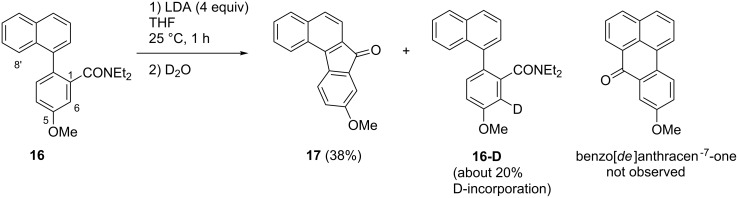
Outcome of a D_2_O quenching experiment after metalation of amide **16** with LDA.

Incubation of methyl ester **15** with LDA (4 equiv LDA, 25 °C) over a period of 1 h, followed by D_2_O quenching resulted in complete decomposition. In contrast, metalation of amide **16** under the same conditions and quenching with D_2_O led to the formation of the benzo[*c*]fluoren-7-one **17** in 38% yield. In recovered (about 20%) educt **16**, deuterium incorporation (about 20%; calculated from integrals of the ^1^H NMR spectrum) was exclusively observed at C-6 of the benzamide moiety (Scheme 8). These data show no indication for a DreM at the *peri*-position C-8’, since neither a 8’-deuterated arylnaphthalene nor a benzo[*de*]anthracen-7-one was detected. This observation is in accordance with results from the Snieckus group [36] on the remote metalation/cyclization of a 2-(1-naphthyl)benzamide, which gave exclusively benzo[*c*]fluorenone (analogue of **17** lacking the methoxy group), and not the benzo[*de*]anthracen-7-one. This clearly indicates that remote metalation, if any, occurs only in the *ortho*-position to the benzamide residue (C-2 of the naphthalene ring system), but not in the *peri*-position (C-8 of the naphthalene). Consequently, further attempts to initialize the cyclization of arylisoquinolines **10c** and **12** to the oxoisoaporphine alkaloid bianfugecine (**6**, Scheme 5) by a DreM did not appear to be promising.

**Scheme 9:**
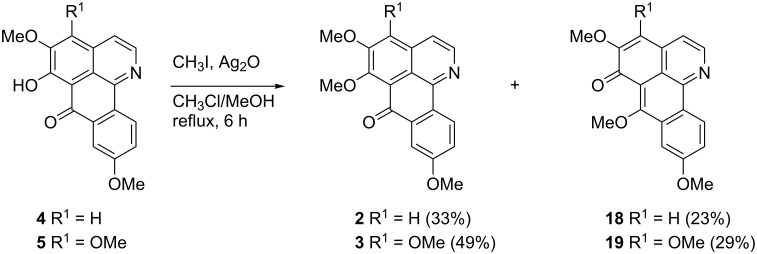
Synthesis of the alkaloids menisporphine (**2**) and dauriporphine (**3**) by *O*-methylation of the alkaloids 6-*O*-demethylmenisporphine (**4**) and dauriporphinoline (**5**).

Finally, the alkaloids 6-*O*-demethylmenisporphine (**4**) and dauriporphinoline (**5**) were converted into their corresponding 6-methoxy derivatives, the oxoisoaporphine alkaloids menisporphine (**2**) and dauriporphine (**3**). Using Kunitomo’s [7,17] protocol (methyl iodide in the presence of silver oxide), phenols **4** and **5** were converted into the alkaloids **2** and **3** in 33 and 49% yields (Scheme 9). In accordance to Kunitomo’s observations, the isomeric methoxy compounds **18** and **19** were obtained as byproducts in significant yields (Scheme 9). In order to find a more convenient methylation protocol, we explored diazomethane, but no conversion was observed at all.

Finally, all of the synthesized alkaloids were tested for their cytotoxic potential in a MTT assay on the HL-60 cell line. Except for menisporphine (**2**) and bianfugecine (**6**, both IC_50_ values > 50 µM), all of the described oxoisoaporphine alkaloids and the isomers **18** and **19** showed significant cytotoxic activity. IC_50_ values of alkaloid **3** and of compounds **18** and **19** are ranging from 3–6 µM. Very strong cytotoxicity was determined for 6-*O*-demethylmenisporphine (**4**) with an IC_50_ value of 0.06 µM and for dauriporphinoline (**5**) with 0.23 µM. So, at least in the HL-60 cell line, oxoisoaporphines bearing a free hydroxy group at C-6 show outstanding cytotoxicity. These data are in accordance with previously reported results [37].

## Conclusion

In conclusion, we worked out a novel approach for the synthesis of oxoisoaporphine alkaloids by direct ring metalation of alkoxy isoquinolines at C-1, followed by the reaction with iodine as central step. Subsequent Suzuki cross-coupling to methyl 2-(isoquinolin-1-yl)benzoates and intramolecular acylation with Eaton’s reagent afforded five alkaloids of the oxoisoaporphine type. Significant cytotoxicity was found for oxoisoaporphines bearing a free 6-hydroxy group.

## Experimental

For experimental procedures and copies of ^1^H and ^13^C NMR spectra of all compounds see Supporting Information File 1.

## Supporting Information

File 1 Experimental procedures and copies of ^1^H and ^13^C NMR spectra.

## References

[1] Hagel J M, Facchini P J (2013). Plant Cell Physiol.

[2] Shamma M, Guinaudeau H (1984). Nat Prod Rep.

[3] Stévigny C, Bailly C, Quetin-Leclercq J (2005). Curr Med Chem - Anti-Cancer Agents.

[4] Kunitomo J, Satoh M (1982). Chem Pharm Bull.

[5] Tang H, Wang X-D, Wie Y-B, Huang S-L, Huang Z-S, Tan J-H, An L-K, Wu J-Y, Chan A S-C, Gu L-Q (2008). Eur J Med Chem.

[6] Castro-Castillo V, Suárez-Rozas C, Pabón A, Pérez E G, Cassels B K, Blair S (2013). Bioorg Med Chem Lett.

[7] Kunitomo J, Satoh M, Shingu T (1983). Tetrahedron.

[8] Okamoto Y, Tanaka S, Kitayama K, Isomoto M, Masaishi M, Yanagawa H, Kunitomo J-I (1971). Yakugaku Zasshi.

[9] Kupchan S M, Moniot J L, Kanojia R M, O'Brien J B (1971). J Org Chem.

[10] Kupchan S M, O’Brien P F (1973). J Chem Soc, Chem Commun.

[11] Castedo L, Saá J M, Suau R, Villaverde C (1980). Heterocycles.

[12] Kessar S V, Gupta Y P, Yadav V S, Narula M, Mohammed T (1980). Tetrahedron Lett.

[13] Melzer B, Bracher F (2015). Org Biomol Chem.

[14] Orito K, Uchiito S, Satoh Y, Tatsuzawa T, Harada R, Tokuda M (2000). Org Lett.

[15] Cuny G D (2004). Tetrahedron Lett.

[16] Chaudhary S, Pecic S, LeGendre O, Harding W W (2009). Tetrahedron Lett.

[17] Kunitomo J, Kaede S, Satoh M (1985). Chem Pharm Bull.

[18] Jia X, Yang D, Zhang S, Cheng J (2009). Org Lett.

[19] Chaitanya M, Yadagiri D, Anbarasan P (2013). Org Lett.

[20] Zhang J, Chen J, Zhang X, Lei X (2014). J Org Chem.

[21] Melzer B, Plodek A, Bracher F (2014). J Org Chem.

[22] Kraskovskiy A, Kraskovskaya V, Knochel P (2006). Angew Chem, Int Ed.

[23] Metzger A, Schade M A, Knochel P (2008). Org Lett.

[24] Krätzschmar F, Kaßel M, Delony D, Breder A (2015). Chem – Eur J.

[25] Plodek A, Raeder S, Bracher F (2013). Tetrahedron.

[26] Bracher F (1989). Arch Pharm.

[27] Mink K, Bracher F (2007). Arch Pharm.

[28] Dorow R L, Herrinton P M, Hohler R A, Maloney M T, Mauragis M A, McGhee W E, Moeslein J A, Strohbach J W, Veley M F (2006). Org Process Res Dev.

[29] Zewge D, Chen C-y, Deer C, Dormer P G, Hughes D L (2007). J Org Chem.

[30] Kunitomo J-i, Miyata Y (1986). Heterocycles.

[31] MacNeil S L, Gray M, Gusev D G, Briggs L E, Snieckus V (2008). J Org Chem.

[32] Levin J I, Turos E, Weinreb S M (1982). Synth Commun.

[33] Snieckus V (1990). Chem Rev.

[34] Tilly D, Magolan J, Mortier J (2012). Chem – Eur J.

[35] Tilly D, Fu J-m, Zhao B-p, Alessi M, Castanet A S, Snieckus V, Mortier J (2010). Org Lett.

[36] Fu J M, Zhao B P, Sharp M J, Snieckus V (1991). J Org Chem.

[37] Cheng J-J, Tsai T-H, Lin L-C (2012). Planta Med.

