# Supplementary material for: A novel approach to oxoisoaporphine alkaloids via regioselective metalation of alkoxy isoquinolines
Source: Beilstein J Org Chem. 2017 Aug 8;13:1564–71. doi: 10.3762/bjoc.13.156 (PMC5564254; doi:10.3762/bjoc.13.156)
Supplement: File 1 — Experimental procedures and copies of 1H and 13C NMR spectra. [file Beilstein_J_Org_Chem-13-1564-s001.pdf]

Supporting Information

for

A novel approach to oxoisoaporphine alkaloids via regioselective metalation of alkoxy isoquinolines

Benedikt C. Melzer and Franz Bracher*

Address: Department für Pharmazie – Zentrum für Pharmaforschung, Ludwig-Maximilians Universität München, Butenandtstr. 5-13, D-81377 Munich, Germany

Email: Franz Bracher* - Franz.Bracher@cup.uni-muenchen.de

*Corresponding author

Experimental procedures and copies of ^{1}H and ^{13}C NMR spectra

Table of contents

Experimental procedures and characterization of all compounds …………………… S3–S16
^{1}H NMR spectrum of **8a**……………………………………………………………….. S17
^{13}C NMR spectrum of **8a**………………………………………………………………. S18
^{1}H NMR spectrum of **8b**……………………………………………………………….. S19
^{13}C NMR spectrum of **8b**………………………………………………………………. S20
^{1}H NMR spectrum of **8c**……………………………………………………………….. S21
^{13}C NMR spectrum of **8c**………………………………………………………………. S22
^{1}H NMR spectrum of **10a**……………………………………………………………… S23
^{13}C NMR spectrum of **10a**……………………………………………………………... S24
^{1}H NMR spectrum of **10b**……………………………………………………………… S25
^{13}C NMR spectrum of **10b**……………………………………………………………... S26
^{1}H NMR spectrum of **10c**……………………………………………………………… S27
^{13}C NMR spectrum of **10c**……………………………………………………………... S28
^{1}H NMR spectrum of **4**………………………………………………………………….. S29
^{13}C NMR spectrum of **4**…………………………………………………………………. S30

^{1}H NMR spectrum of **5**…………………………………………………………………….. S31
^{13}C NMR spectrum of **5**……………………………………………………….…………… S32
^{1}H NMR spectrum of **6**…………………………………………………………………….. S33
^{13}C NMR spectrum of **6**…………………………………………………………………….. S34
^{1}H NMR spectrum of **2**…………………………………………………………………….. S35
^{13}C NMR spectrum of **2**……………………………………………………….…………… S36
^{1}H NMR spectrum of **18**…………………………………………………………………….. S37
^{13}C NMR spectrum of **18**……………………………………………………….…………… S38
^{1}H NMR spectrum of **3**…………………………………………………………………….. S39
^{13}C NMR spectrum of **3**……………………………………………………….…………… S40
^{1}H NMR spectrum of **19**…………………………………………………………………….. S41
^{13}C NMR spectrum of **19**……………………………………………………….…………… S42
^{1}H NMR spectrum of **12**…………………………………………………………………….. S43
^{13}C NMR spectrum of **12**……………………………………………………….…………… S44
^{1}H NMR spectrum of **15**…………………………………………………………………….. S45
^{13}C NMR spectrum of **15**……………………………………………………….…………… S46
^{1}H NMR spectrum of **16**…………………………………………………………………….. S47
^{13}C NMR spectrum of **16**……………………………………………………….…………… S48
^{1}H NMR spectrum of **17**…………………………………………………………………….. S49
^{13}C NMR spectrum of **17**……………………………………………………….…………… S50
Comparison of ^{1}H NMR spectra of **12** and **12-D**…………………………………………… S51
Comparison of ^{1}H NMR spectra of **16** and **16-D**…………………………………………… S52
References……………………………………..…………………………………………… S53

Experimental procedures and characterization of all compounds

General Information

Solvents used were of HPLC grade or p.a. grade and/or purified according to standard procedures. Melting points were determined by open tube capillary method with a Büchi melting point B-450 apparatus. IR measurements were carried out with a Perkin–Elmer FTIR Paragon 1000 spectrometer. NMR spectra were recorded with Jeol J NMR GX (400 or 500 MHz) and Avance III HD Bruker BioSpin (400 or 500 MHz) spectrometers with residual non-deuterated solvent as internal standard. Spectra were recorded in deuterated solvents and chemical shifts are reported in parts per million (ppm). *J* values are given in Hertz. Multiplicities are abbreviated as follows: s = singlet, d = doublet, t = triplet, m = multiplet. Signal assignments were carried out based on ^{1}H, ^{13}C, HMBC, HMQC and COSY spectra. NMR spectra were analyzed with the NMR software MestReNova, Version 5.1.1-3092 (Mestrelab Research S.L.). HRMS were performed by electron impact (EI) at 70 eV with a Thermo Finnigan MAT 95 or a Jeol GCmate II spectrometer or by electrospray ionization (ESI) with a Thermo Finnigan LTQ FT Ultra Fourier Transform Ion Cyclotron resonance mass spectrometer. Chromatographic purification of products was performed by using flash column chromatography on Merck silica gel 60 (0.015–0.040 mm) as stationary phase.

General Procedures

General procedure A (iodination of isoquinolines 7a–c)

A flame-dried and nitrogen flushed 50 mL Schlenk flask, equipped with a magnetic stirring bar, was charged with the appropriate isoquinoline **7a–c** (prepared according to lit. [1,2]) (2.00 mmol) in dry THF (10 mL). TMPMgCl·LiCl (1.0 M in THF/toluene; 3.00 mL, 3.00 mmol) was added to this solution dropwise over 2 min. The reaction mixture was stirred at room temperature for 4 h. After cooling to 0 °C, a solution of iodine (0.761 g, 3.00 mmol) in dry

THF (3 mL) was added dropwise to the reaction mixture. The cooling bath was removed and the mixture was stirred at room temperature for 1 h. Then the mixture was quenched with satd. aqueous NH_4Cl solution (4 mL) and satd. aqueous $Na_2S_2O_3$ solution (4 mL). After extraction with dichloromethane (3 × 20 mL), the combined organic layers were dried with Na_2SO_4, and concentrated under reduced pressure. The residue was purified by flash column chromatography (ethyl acetate/dichloromethane = 1:5).

General procedure B (Suzuki cross-coupling of iodinated isoquinolines 8a–c)

The appropriate 1-iodoisoquinoline **8a-c**, methyl 5-methoxy-2-(4,4,5,5-tetramethyl-1,3,2-dioxaborolan-2-yl)benzoate (**9**) (prepared according to lit. [3]) (1.2 equiv.) and $Pd(PPh_3)_4$ (5 mol %) were dissolved in THF (7–12 mL). After addition of an aqueous K_2CO_3 solution (1.0 M in water; 6.0 equiv) the mixture was heated under nitrogen at reflux for 24 h. The reaction mixture was allowed to cool to room temperature, poured into water (50 mL), and extracted with ethyl acetate (4 × 50 mL). The combined organic layers were dried with Na_2SO_4, and concentrated under reduced pressure. The residue was purified by flash column chromatography.

General procedure C (ester hydrolysis and cyclization with Eaton's reagent to give oxoisoaporphines 4, 5, and 6)

The appropriate methyl ester (**10a–c**) was dissolved in concentrated hydrochloric acid (5–10 mL) and the mixture was heated at reflux for 2.5 h. The reaction mixture was allowed to cool to room temperature and then poured into water (50–100 mL). After neutralization to pH 7 with 10% aqueous KOH solution the mixture was extracted with *n*-butanol (4 × 100–250 mL), and the combined organic layers were concentrated under reduced pressure. To the crude residue Eaton's reagent (purchased from Sigma-Aldrich) (4–5 mL) was added dropwise, and the reaction mixture was stirred at 90 °C for 2 h. The reaction mixture was allowed to cool to room temperature and was then cautiously added dropwise to a 10% aqueous NH_3 solution (50–100 mL) while stirring. The mixture was extracted with dichloromethane (4 × 50–100

mL). The combined organic layers were dried with Na_2SO_4, and concentrated under reduced pressure. The residue was purified by flash column chromatography (dichloromethane/methanol = 97:3).

General procedure D (*O*-methylation of phenolic alkaloids 4 and 5)

The appropriate phenolic alkaloid (**4** or **5**) (0.50 or 0.65 mmol) was suspended in a mixture of chloroform (8 mL) and methanol (6 mL). Ag_2O (7 equiv) and CH_3I (240 equiv) were added and the mixture was heated to reflux for 6 h. After cooling to room temperature, the mixture was filtered and the precipitate was thoroughly washed with chloroform. The filtrate and the chloroform-extract were combined, washed with water (2 × 50 mL), dried with $Na_2SO_{4,}$ and concentrated under reduced pressure. The residue was purified by flash column chromatography (dichloromethane/acetone = 98:2).

General procedure E (conversion of methyl esters to diethyl amides 12 and 16)

A flame-dried and nitrogen flushed 25 mL Schlenk flask, equipped with a magnetic stirring bar, was charged with diethylamine (2.0 equiv) in dry toluene (3 mL). Trimethylaluminium (2.0 M in toluene; 2.0 equiv) was added dropwise at 0 °C. The mixture was stirred at room temperature for 1 h before ester **10c** or **15** (1.0 equiv) in dry toluene (2 mL) was added. The reaction mixture was heated to reflux for 2 h under an atmosphere of nitrogen. After cooling to room temperature the mixture was poured into 1 M HCl (50 mL) and made alkaline (pH 9) by the addition of 6 N NaOH. After extraction with ethyl acetate (3 × 50 mL), the combined organic layers were dried with Na_2SO_4, and concentrated under reduced pressure. The residue was purified by flash column chromatography.

Compounds

1-Iodo-6,7-dimethoxyisoquinoline (8a)

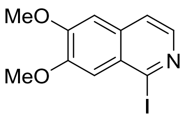

This compound was prepared following general procedure A from 6,7-dimethoxyisoquinoline (**7a**, 0.378 g, 2.00 mmol) with TMPMgCl·LiCl (1.0 M in THF/toluene; 3.0 mL, 3.00 mmol) to give **8a** (0.419 g, 67%) as a brown solid. mp 139 – 140 °C (lit. [4] 140 – 141 °C); ^{1}H NMR (500 MHz, $CDCl_3$): δ (ppm) = 8.11 (d, *J* = 5.5 Hz, 1H), 7.42 (d, *J* = 5.5 Hz, 1H), 7.36 (s, 1H), 7.00 (s, 1H), 4.07 (s, 3H), 4.04 (s, 3H); ^{13}C NMR (126 MHz, $CDCl_3$): δ (ppm) = 153.6, 151.6, 142.1, 132.7, 128.2, 124.9, 120.2, 111.2, 105.2, 56.5, 56.4; HRMS (EI): *m/z* (%) = 314.9801 (calcd for $C_{11}H_{10}INO_2$: 314.9757); IR (KBr pellet): ν (cm^{-1}) = 2974, 2933, 2834, 1616, 1582, 1554, 1508, 1393, 1253, 1227, 1145, 1006, 930, 860, 775, 672.

1-Iodo-5,6,7-trimethoxyisoquinoline (8b)

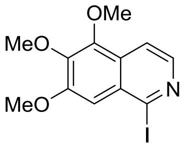

This compound was prepared following general procedure A from 5,6,7-trimethoxyisoquinoline (**7b**, 0.438 g, 2.00 mmol) with TMPMgCl·LiCl (1.0 M in THF/toluene; 3.0 mL, 3.00 mmol) to give **8b** (0.365 g, 53%) as a brown solid. mp 86 – 88 °C; ^{1}H NMR (400 MHz, $CDCl_3$): δ (ppm) = 8.13 (d, *J* = 5.6 Hz, 1H), 7.77 (dd, *J* = 5.6, 0.7 Hz, 1H), 7.20 (s, 1H), 4.05 (s, 3H), 4.03 (s, 3H), 4.02 (s, 3H); ^{13}C NMR (101 MHz, $CDCl_3$): δ (ppm) = 154.8, 146.6, 144.5, 141.4, 129.2, 127.9, 125.1, 115.5, 107.5, 61.8, 61.4, 56.4; HRMS (EI): *m/z* (%) = 344.9856 (calcd for $C_{12}H_{12}INO_3$: 344.9862); IR (KBr pellet): ν (cm^{-1}) = 2966, 2936, 2835, 2360, 2342, 1615, 1576, 1549, 1484, 1471, 1427, 1376, 1350, 1299, 1254, 1240, 1201, 1180, 1136, 1118, 1034, 997, 944, 904, 829, 732, 651.

1-Iodo-6-methoxyisoquinoline (8c)

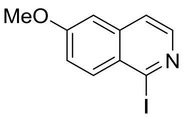

This compound was prepared following general procedure A from 6-methoxyisoquinoline (**7c**, 0.318 g, 2.00 mmol) with TMPMgCl·LiCl (1.0 M in THF/toluene; 3.0 mL, 3.00 mmol) to give **8c** (0.336 g, 59%) as a brown solid. mp 53 – 54 °C; ^{1}H NMR (400 MHz, $CDCl_3$): δ (ppm) = 8.12 (d, *J* = 5.6 Hz, 1H), 7.96 (d, *J* = 9.3 Hz, 1H), 7.43 (d, *J* = 5.6 Hz, 1H), 7.24 (dd, *J* = 9.3, 2.5 Hz, 1H), 6.96 (d, *J* = 2.5 Hz, 1H), 3.93 (s, 3H); ^{13}C NMR (101 MHz, $CDCl_3$): δ (ppm) = 161.5, 143.5, 138.1, 134.8, 127.6, 126.7, 121.7, 120.7, 104.7, 55.8; HRMS (EI): *m/z* (%) = 284.9644 (calcd for $C_{10}H_8INO$: 284.9651); IR (KBr pellet): ν (cm^{-1}) = 3004, 2925, 2829, 2360, 2343, 1619, 1555, 1486, 1467, 1432, 1396, 1373, 1339, 1301, 1261, 1241, 1168, 1133, 1029, 958, 864, 814, 695, 657.

Methyl 2-(6,7-dimethoxyisoquinolin-1-yl)-5-methoxybenzoate (10a)

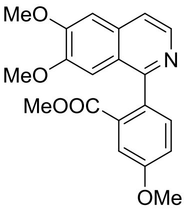

This compound was prepared following general procedure B from 1-iodo-6,7-dimethoxyisoquinoline (**8a**) (0.315 g, 1.00 mmol), methyl 5-methoxy-2-(4,4,5,5-tetramethyl-1,3,2-dioxaborolan-2-yl)benzoate (**9**) (0.351 g, 1.20 mmol), $Pd(PPh_3)_4$ (0.058 g, 0.05 mmol). The residue was purified by flash column chromatography (dichloromethane/methanol = 97 : 3) to give **10a** (0.272 g, 77%) as a white solid. mp 149 – 151 °C (lit. [5] 122 – 123 °C); ^{1}H NMR (400 MHz, CD_2Cl_2): δ (ppm) = 8.36 (d, *J* = 5.6 Hz, 1H), 7.52 (d, *J* = 2.8 Hz, 1H), 7.49 (d, *J* = 5.6 Hz, 1H), 7.46 (d, *J* = 8.6 Hz, 1H), 7.19 (dd, *J* = 8.6, 2.8 Hz, 1H), 7.14 (s, 1H), 6.94 (s, 1H), 3.99 (s, 3H), 3.93 (s, 3H), 3.75 (s, 3H), 3.41 (s, 3H); ^{13}C NMR (101 MHz, CD_2Cl_2): δ (ppm) = 168.1, 159.9, 158.5, 153.2, 150.7, 141.6, 133.8, 133.5, 133.3, 132.6, 123.8, 119.0, 118.0, 115.4, 105.5, 105.4, 56.4, 56.2 (2C), 52.4; HRMS (EI): *m/z* (%) = 353.1258 (calcd for

$C_{20}H_{19}NO_5$: 353.1263); IR (KBr pellet): ν (cm^{-1}) = 3071, 3012, 2965, 2943, 2838, 2360, 2342, 1714, 1618, 1568, 1508, 1480, 1435, 1414, 1351, 1319, 1291, 1235, 1161, 1127, 1084, 1038, 887, 864, 831, 799.

Methyl 5-methoxy-2-(5,6,7-trimethoxyisoquinolin-1-yl)benzoate (10b)

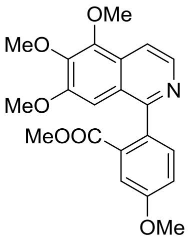

This compound was prepared following general procedure B from 1-iodo-5,6,7-trimethoxyisoquinoline (**8b**) (0.219 g, 0.63 mmol), methyl 5-methoxy-2-(4,4,5,5-tetramethyl-1,3,2-dioxaborolan-2-yl)benzoate (**9**) (0.222 g, 0.76 mmol), $Pd(PPh_3)_4$ (0.035 g, 0.03 mmol). The residue was purified by flash column chromatography (dichloromethane/ethyl acetate = 1 : 1) to give **10b** (0.157 g, 65%) as slightly yellow needles. mp 134 – 135 °C (lit. [6] 131.5 – 134.5 °C); ^{1}H NMR (400 MHz, CD_2Cl_2): δ (ppm) = 8.39 (d, J = 5.8 Hz, 1H), 7.80 (dd, J = 5.8, 0.8 Hz, 1H), 7.52 (d, J = 2.8 Hz, 1H), 7.43 (d, J = 8.5 Hz, 1H), 7.19 (dd, J = 8.5, 2.8 Hz, 1H), 6.77 (s, 1H), 4.06 (s, 3H), 3.97 (s, 3H), 3.93 (s, 3H), 3.75 (s, 3H), 3.43 (s, 3H); ^{13}C NMR (101 MHz, CD_2Cl_2): δ (ppm) = 168.0, 160.0, 158.8, 154.1, 147.4, 144.2, 141.0, 133.7, 133.2, 132.6, 128.6, 125.1, 118.0, 115.5, 114.1, 101.6, 62.0, 61.5, 56.3, 56.2, 52.4; HRMS (EI): *m/z* (%) = 383.1364 (calcd for $C_{21}H_{21}NO_6$: 383.1369); IR (KBr pellet): ν (cm^{-1}) = 3008, 2938, 2840, 2360, 2342, 1713, 1614, 1572, 1556, 1488, 1476, 1443, 1405, 1376, 1297, 1241, 1193, 1126, 1070, 1041, 1028, 1001, 956, 868, 835.

Methyl 5-methoxy-2-(6-methoxyisoquinolin-1-yl)benzoate (10c)

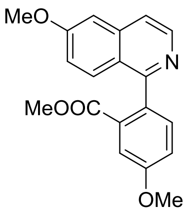

This compound was prepared following general procedure B from 1-iodo-6-methoxyisoquinoline (**8c**) (0.360 g, 1.26 mmol), methyl 5-methoxy-2-(4,4,5,5-tetramethyl-1,3,2-dioxaborolan-2-yl)benzoate (**9**) (0.442 g, 1.51 mmol), $Pd(PPh_3)_4$ (0.073 g, 0.06 mmol). The residue was purified by flash column chromatography (dichloromethane/methanol = 97 : 3) to give **10c** (0.304 g, 75%) as a slightly yellow solid. mp 76 – 77 °C; ^{1}H NMR (500 MHz, $CDCl_3$): δ (ppm) = 8.47 (d, J = 5.7 Hz, 1H), 7.59 (d, J = 2.7 Hz, 1H), 7.57 – 7.53 (m, 2H), 7.41 (d, J = 8.4 Hz, 1H), 7.18 (dd, J = 8.4, 2.7 Hz, 1H), 7.11 (d, J = 2.5 Hz, 1H), 7.08 (dd, J = 9.0, 2.5 Hz, 1H), 3.95 (s, 3H), 3.93 (s, 3H), 3.43 (s, 3H); ^{13}C NMR (126 MHz, $CDCl_3$): δ (ppm) = 167.3, 160.6, 160.3, 159.6, 142.6, 138.3, 133.3, 132.2, 132.1, 128.9, 123.5, 120.0, 119.3, 118.2, 115.1, 104.6, 55.8, 55.6, 52.1; HRMS (EI): *m/z* (%) = 323.1154 (calcd for $C_{19}H_{17}NO_4$: 323.1158); IR (KBr pellet): ν (cm^{-1}) = 3055, 2953, 2835, 1714, 1621, 1560, 1507, 1468, 1437, 1406, 1375, 1357, 1313,1256, 1233, 1222, 1120, 1077, 1029, 986, 973, 858, 840, 793, 782, 680.

6-Hydroxy-5,9-dimethoxy-7*H*-dibenzo[*de,h*]quinolin-7-one

(4, 6-*O*-demethylmenisporphine)

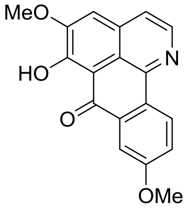

This compound was prepared following general procedure C from methyl 2-(6,7-dimethoxyisoquinolin-1-yl)-5-methoxybenzoate (**10a**) (0.611 g, 1.73 mmol) using conc. HCl (10 mL), H_2O (100 mL), and for extraction *n*-butanol (4 × 250 mL). The crude acid was further treated following the general procedure with Eaton's reagent (5 mL), aqueous NH_3 solution

(100 mL) and for extraction dichloromethane (4 × 100 mL) to give **4** (0.239 g, 45%) as a yellow solid. mp 245 – 246 °C (lit. [5] 248 – 249 °C); ^{1}H NMR (500 MHz, $CDCl_3$): δ (ppm) = 8.98 (d, *J* = 8.9 Hz, 1H), 8.77 (d, *J* = 5.2 Hz, 1H), 7.95 (d, *J* = 2.7 Hz, 1H), 7.61 (d, *J* = 5.2 Hz, 1H), 7.47 (dd, *J* = 8.9, 2.7 Hz, 1H), 7.33 (s, 1H), 4.11 (s, 3H), 4.02 (s, 3H); ^{13}C NMR (126 MHz, $CDCl_3$): δ (ppm) = 184.8, 164.7, 161.0, 153.0, 144.2, 143.9, 131.9, 131.2, 130.8, 127.3, 123.4, 119.9, 116.4, 112.3, 109.0, 107.4, 56.5, 55.9; HRMS (EI): *m/z* (%) = 307.0829 (calcd for $C_{18}H_{13}NO_4$: 307.0845); IR (KBr pellet): ν (cm^{-1}) = 3422, 2978, 2841, 2360, 2342, 1584, 1494, 1481, 1438, 1278, 1202, 1028, 1003, 866, 840, 818, 668, 623.

6-Hydroxy-4,5,9-trimethoxy-7*H*-dibenzo[*de,h*]quinolin-7-one (5, dauriporphinoline)

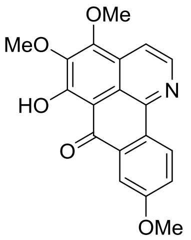

This compound was prepared following general procedure C from methyl 2-(5,6,7-trimethoxyisoquinolin-1-yl)-5-methoxybenzoate (**10b**) (0.339 mg, 0.88 mmol) using conc. HCl (5 mL), H_2O (50 mL), and for extraction *n*-butanol (4 × 100 mL). The crude acid was further treated following the general procedure with Eaton's reagent (5 mL), aqueous NH_3 solution (50 mL) and for extraction dichloromethane (4 × 50 mL) to give **5** (0.173 g, 58%) as a yellow solid. mp 196 – 197 °C; ^{1}H NMR (500 MHz, $CDCl_3$): δ = 9.00 (d, *J* = 8.9 Hz, 1H), 8.81 (d, *J* = 5.3 Hz, 1H), 7.99 (d, *J* = 5.3 Hz, 1H), 7.95 (d, *J* = 2.7 Hz, 1H), 7.45 (dd, *J* = 8.9, 2.7 Hz, 1H), 4.36 (s, 3H), 4.10 (s, 3H), 4.02 (s, 3H); ^{13}C NMR (126 MHz, $CDCl_3$): δ = 181.9, 170.7, 161.0, 154.8, 143.9, 143.8, 140.7, 131.9, 130.6, 127.4, 127.2, 122.8, 117.6, 116.1, 107.1, 106.1, 61.8, 61.7, 55.9; HRMS (EI): *m/z* (%) = 337.0940 (calcd for $C_{19}H_{15}NO_5$: 337.0950); IR (KBr pellet): ν (cm^{-1}) = 3448, 2948, 2366, 2345, 1611, 1577, 1560, 1486, 1457, 1397, 1375, 1288, 1145, 1090, 1073, 1014, 980, 829, 741, 638.

5,9-Dimethoxy-7*H*-dibenzo[*de,h*]quinolin-7-one (6, bianfugecine)

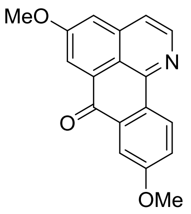

This compound was prepared following general procedure C from methyl 5-methoxy-2-(6-methoxyisoquinolin-1-yl)benzoate (**10c**) (0.120 g, 0.37 mmol) using conc. HCl (5 mL), H_2O (50 mL), and for extraction *n*-butanol (4 × 100 mL). The crude acid was further treated following the general procedure with Eaton's reagent (4 mL), aqueous NH_3 solution (50 mL) and for extraction dichloromethane (4 × 50 mL) to give **6** (0.008 g, 7%) as a yellow solid. mp 196 – 198 °C (lit. [7] 199 – 201 °C); ^{1}H NMR (400 MHz, $CDCl_3$): δ (ppm) = 8.80 (d, *J* = 8.7 Hz, 1H), 8.65 (d, *J* = 5.7 Hz, 1H), 8.27 (d, *J* = 2.6 Hz, 1H), 7.85 (d, *J* = 2.8 Hz, 1H), 7.59 (d, *J* = 5.7 Hz, 1H), 7.41 (d, *J* = 2.6 Hz, 1H), 7.35 (dd, *J* = 8.8, 2.8 Hz, 1H), 4.04 (s, 3H), 3.99 (s, 3H); HRMS (EI): *m/z* (%) = 291.0895 (calcd for $C_{18}H_{13}NO_3$: 291.0895).

5,6,9-Trimethoxy-7*H*-dibenzo[*de,h*]quinolin-7-one (2, menisporphine)

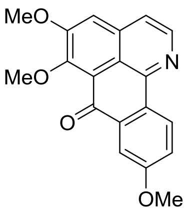

This compound was prepared following general procedure D from 6-hydroxy-5,9-dimethoxy-7*H*-dibenzo[*de,h*]quinolin-7-one (**4**) (0.200 g, 0.65 mmol), Ag_2O (1.054 g, 4.55 mmol), and CH_3I (9.7 mL, 156 mmol) to give **2** (0.070 g, 33%) as yellow needles. mp 185 – 186 °C (lit. [5] 199.5 – 200.5 °C); ^{1}H NMR (400 MHz, $CDCl_3$): δ (ppm) = 8.78 (d, *J* = 8.8 Hz, 1H), 8.65 (d, *J* = 5.5 Hz, 1H), 7.86 (d, *J* = 2.8 Hz, 1H), 7.55 (d, *J* = 5.5 Hz, 1H), 7.39 (s, 1H), 7.33 (dd, *J* = 8.8, 2.8 Hz, 1H), 4.14 (s, 3H), 4.07 (s, 3H), 3.98 (s, 3H); ^{13}C NMR (101 MHz, $CDCl_3$): δ (ppm) = 182.8, 161.5, 156.6, 155.7, 147.5, 143.8, 135.0, 133.4, 129.8, 127.0, 122.1, 120.6, 119.2, 118.6, 111.6, 109.1, 61.7, 56.4, 55.8; HRMS (EI): *m/z* (%) = 321.0996 (calcd for $C_{19}H_{15}NO_4$:

321.1001); IR (KBr pellet): ν (cm^{-1}) = 3422, 2965, 2361, 1656, 1604, 1474, 1413, 1349, 1279, 1242, 1140, 1027, 1013, 992, 864, 843, 628, 607.

The isomer **5,7,9-trimethoxy-6*H*-dibenzo[*de,h*]quinolin-6-one (18)**

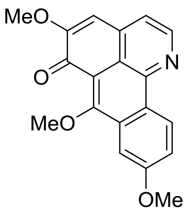

was obtained as yellow needles (0.049 g, 23%). mp 168 – 169 °C (lit. [5] 173 – 175 °C); ^{1}H NMR (500 MHz, $CDCl_3$): δ (ppm) = 9.18 (d, *J* = 9.0 Hz, 1H), 8.87 (d, *J* = 4.6 Hz, 1H), 7.86 (d, *J* = 2.6 Hz, 1H), 7.50 (dd, *J* = 9.0, 2.6 Hz, 1H), 7.44 (d, *J* = 4.6 Hz, 1H), 6.77 (s, 1H), 4.22 (s, 3H), 4.02 (s, 3H), 3.98 (s, 3H); ^{13}C NMR (126 MHz, $CDCl_3$): δ (ppm) = 177.9, 164.2, 160.5, 156.1, 147.9, 142.1, 135.1, 131.8, 130.3, 126.8, 122.2, 119.9, 117.8, 115.5, 109.2, 104.8, 62.7, 56.1, 55.8; HRMS (EI): *m/z* (%) = 321.0995 (calcd for $C_{19}H_{15}NO_4$: 321.1001); IR (KBr pellet): ν (cm^{-1}) = 3430, 2937, 1648, 1624, 1519, 1455, 1418, 1307, 1286, 1267, 1225, 1200, 1138, 1090, 1029, 985, 838.

4,5,6,9-Tetramethoxy-7*H*-dibenzo[*de,h*]quinolin-7-one (3, dauriporphine)

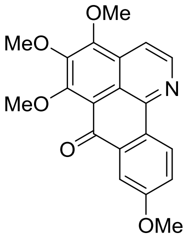

This compound was prepared following general procedure D from 6-hydroxy-4,5,9-trimethoxy-7*H*-dibenzo[*de,h*]quinolin-7-one (**5**) (0.170 g, 0.50 mmol), Ag_2O (0.811 g, 3.50 mmol, and CH_3I (7.5 mL, 120 mmol) to give **3** (0.086 g, 49%) as yellow needles. mp 159 – 161 °C (lit. [6] 161 – 163 °C); ^{1}H NMR (500 MHz, $CDCl_3$): δ (ppm) = 8.81 (d, *J* = 8.7 Hz, 1H), 8.68 (d, *J* = 5.6 Hz, 1H), 7.95 (d, *J* = 5.6 Hz, 1H), 7.88 (d, *J* = 2.7 Hz, 1H), 7.32 (dd, *J* = 8.7, 2.7 Hz, 1H), 4.27 (s, 3H), 4.17 (s, 3H), 4.05 (s, 3H), 3.98 (s, 3H); ^{13}C NMR (126 MHz, $CDCl_3$) δ (ppm) = 181.6, 161.6, 161.0, 153.2, 147.5, 146.5, 143.2, 135.2, 129.6, 128.6, 127.1, 121.8,

120.2, 116.6, 114.6, 109.0, 62.0 (2C), 61.9, 55.8; HRMS (EI): *m/z* (%) = 351.1099 (calcd for $C_{20}H_{17}NO_5$: 351.1107); IR (KBr pellet): ν (cm^{-1}) = 3448, 2946, 2363, 2345, 1645, 1602, 1572, 1487, 1467, 1458, 1396, 1352, 1336, 1280, 1214, 1126, 1022, 830, 734, 636.

The isomer **4,5,7,9-tetramethoxy-6*H*-dibenzo[*de,h*]quinolin-6-one (19)**

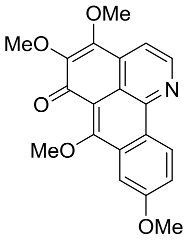

was obtained as yellow needles (0.051 g, 29%). mp 194 – 196 °C (lit. [6] 159 – 161.5 °C); ^{1}H NMR (500 MHz, $CDCl_3$): δ (ppm) = 9.20 (d, *J* = 9.0 Hz, 1H), 8.97 (d, *J* = 4.8 Hz, 1H), 7.94 (d, *J* = 4.8 Hz, 1H), 7.85 (d, *J* = 2.7 Hz, 1H), 7.50 (dd, *J* = 9.0, 2.7 Hz, 1H), 4.30 (s, 3H), 4.22 (s, 3H), 4.03 (s, 3H), 4.02 (s, 3H); ^{13}C NMR (126 MHz, $CDCl_3$): δ (ppm) = 180.3, 162.9, 160.6, 152.3, 147.9, 143.0, 142.3, 133.5, 131.9, 129.8, 126.9, 121.7, 117.9, 116.9, 115.5, 104.9, 62.6, 61.2, 61.1, 55.8.; HRMS (EI): *m/z* (%) = 351.1108 (calcd for $C_{20}H_{17}NO_5$: 351.1107); IR (KBr pellet): ν (cm^{-1}) = 3440. 2936, 1622, 1615, 1560, 1521, 1455, 1419, 1373, 1355, 1302, 1271, 1228, 1204, 1127, 1088, 1069, 1019, 989, 935, 830.

***N*,*N*-Diethyl-5-methoxy-2-(6-methoxyisoquinolin-1-yl)benzamide (12)**

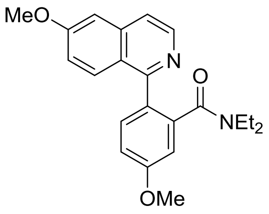

This compound was prepared following general procedure E from diethylamine (0.082 g, 1.12 mmol), trimethylaluminium (2.0 M in toluene; 0.56 mL, 1.12 mmol) and methyl 5-methoxy-2-(6-methoxyisoquinolin-1-yl)benzoate (**10c**) (0.181 mg, 0.56 mmol). The residue was purified by flash column chromatography (ethyl acetate/dichloromethane = 2 : 1) to give **12** (0.064 g, 31%) as a white solid. mp 156 – 157 °C; ^{1}H NMR (500 MHz, CD_2Cl_2): δ (ppm) = 8.38 (d, *J* = 5.7 Hz, 1H), 7.81 – 7.78 (m, 1H), 7.51 (d, *J* = 6.3 Hz, 1H), 7.42 (d, *J* = 8.5 Hz,

1H), 7.13 – 7.11 (m, 2H), 7.04 (dd, *J* = 8.5, 2.7 Hz, 1H), 6.96 (d, *J* = 2.6 Hz, 1H), 3.94 (s, 3H), 3.90 (s, 3H), 3.45 – 2.86 (m_{br}, 4H, 2xCH_2), 0.95 (t, *J* = 7.1 Hz, 3H), 0.59 (t, *J* = 7.1 Hz, 3H); ^{13}C NMR (126 MHz, CD_2Cl_2): δ (ppm) = 170.0, 161.2, 160.1, 159.1, 142.9, 140.2, 139.0, 132.2, 130.1, 129.9, 123.6, 120.1, 119.7, 114.3, 112.4, 104.9, 56.1, 56.0, 43.4, 38.4, 14.0, 12.0; HRMS (EI): *m/z* (%) = 364.1752 (calcd for $C_{22}H_{24}N_2O_3$: 364.1787); IR (KBr pellet): ν (cm^{-1}) = 2963, 2933, 2361, 1625, 1601, 1560, 1475, 1376, 1361, 1269, 1234, 1128, 1029, 974, 880, 868, 826.

Methyl 5-methoxy-2-(naphthalen-1-yl)benzoate (15)

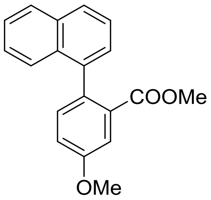

Methyl 2-bromo-5-methoxybenzoate (**14**) (0.980 g, 4.00 mmol), naphthalene-1-boronic acid (**13**) (0.826 g, 4.80 mmol) and $Pd(PPh_3)_4$ (0.231 g, 0.20 mmol) were dissolved in THF (24 mL). After addition of an aqueous K_2CO_3 solution (1.0 M in water; 12.0 mL, 12.00 mmol) the mixture was heated under nitrogen at reflux for 16 h. The reaction mixture was allowed to cool to room temperature, poured into water (50 mL), and extracted with ethyl acetate (3 × 50 mL). The combined organic layers were dried with Na_2SO_4, and concentrated under reduced pressure. The residue was purified by flash column chromatography (*i*-hexane/ethyl acetate = 9 : 1) to give **15** (0.791 g, 68%) as a white solid. mp 129 – 130 °C; ^1H NMR (400 MHz, CD_2Cl_2): δ (ppm) = 7.90 (d, *J* = 8.1 Hz, 1H), 7.85 (d, *J* = 8.3 Hz, 1H), 7.54 (d, *J* = 2.8 Hz, 1H), 7.52 – 7.44 (m, 3H), 7.37 (ddd, *J* = 8.3, 6.8, 1.3 Hz, 1H), 7.33 – 7.27 (m, 2H), 7.17 (dd, *J* = 8.4, 2.8 Hz, 1H), 3.92 (s, 3H), 3.39 (s, 3H); ^{13}C NMR (101 MHz, CD_2Cl_2): δ (ppm) = 167.9, 159.5, 140.1, 134.1, 133.9, 133.6, 133.0, 133.0, 128.7, 127.8, 126.8, 126.4, 126.1, 126.1, 125.7, 118.1, 115.3, 56.2, 52.2; HRMS (EI): *m/z* (%) = 292.1086 (calcd for $C_{19}H_{16}O_3$: 292.1099); IR (KBr pellet): ν (cm^{-1}) = 3015, 2950, 1719, 1602, 1559, 1495, 1430, 1395, 1283, 1248, 1214, 1118, 1075, 1055, 1033, 881, 834, 806, 787.

_N,N_-Diethyl-5-methoxy-2-(napththalen-1-yl)benzamide (16)

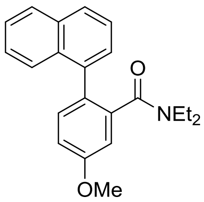

This compound was prepared following general procedure E from diethylamine (0.100 g, 1.37 mmol), trimethylaluminium (2.0 M in toluene; 0.68 mL, 1.37 mmol) and methyl 5-methoxy-2-(naphthalen-1-yl)benzoate (**15**) (0.200 mg, 0.68 mmol). The residue was purified by flash column chromatography (ethyl acetate/*i*-hexane = 1 : 1) to give **16** (0.133 g, 58%) as a pale white solid. mp 107 – 109 °C; ^{1}H NMR (400 MHz, $(CD_3)_2SO$, 333 K): δ (ppm) = 7.93 (d, *J* = 8.0 Hz, 1H), 7.89 (d, *J* = 8.2 Hz, 1H), 7.65 (d, *J* = 8.1 Hz, 1H), 7.52 – 7.41 (m, 3H), 7.35 (d, *J* = 6.9 Hz, 1H), 7.32 (d, *J* = 8.5 Hz, 1H), 7.11 (dd, *J* = 8.5, 2.7 Hz, 1H), 6.95 (d, *J* = 2.7 Hz, 1H), 3.87 (s, 3H), 3.16 – 2.57 (m_{br}, 4H), 0.95 – 0.54 (m, 3H), 0.42 (t, *J* = 7.0 Hz, 3H); ^{13}C NMR (101 MHz, $(CD_3)_2SO$, 333K): δ (ppm) = 168.0, 158.4, 138.8, 136.3, 133.0, 131.9, 131.4, 128.3, 127.8, 127.3, 127.0, 125.5, 125.3, 124.6 (2C), 113.7, 111.5, 55.2, 41.8, 36.9, 13.1, 11.2; HRMS (EI): *m/z* (%) = 333.1719 (calcd for $C_{22}H_{23}NO_2$: 333.1729); IR (KBr pellet): ν (cm^{-1}) = 3056, 2970, 2933, 2836, 1630, 1607, 1564, 1473, 1460, 1432, 1393, 1314, 1290, 1271, 1230, 1169, 1074, 1035, 803, 780.

9-Methoxy-7*H*-benzo[*c*]fluoren-7-one (17)

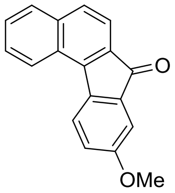

A flame-dried and nitrogen flushed 10 mL Schlenk tube, equipped with a magnetic stirring bar, was charged with *N*,*N*-diethyl-5-methoxy-2-(napththalen-1-yl)benzamide (**16**) (0.060 g, 0.18 mmol) in dry THF (2 mL). LDA (2.0 M in THF/heptane/ethylbenzene; 0.36 mL, 0.72 mmol) was added to this solution dropwise at 0 °C. The mixture was stirred at room temperature for 1 h. Then the mixture was quenched with deuterium oxide (0.3 mL) and

stirred for 20 min. Satd. aqueous NH_4Cl solution (3 mL) was added and the mixture was extracted with dichloromethane (3 × 20 mL). The combined organic layers were dried with Na_2SO_4 and concentrated under reduced pressure. The residue was purified by flash column chromatography (ethyl acetate/*i*-hexane = 1 : 1) to give **17** (0.018 g, 38%) as a red solid. mp 133 – 134 °C; ^1H NMR (500 MHz, CD_2Cl_2): δ (ppm) = 8.42 – 8.39 (m, 1H), 7.89 (d, *J* = 8.3 Hz, 1H), 7.88 – 7.85 (m, 1H), 7.70 (d, *J* = 8.2 Hz, 1H), 7.63 (d, *J* = 8.2 Hz, 1H), 7.62 – 7.55 (m, 2H), 7.20 – 7.19 (m, 1H), 6.98 (dd, *J* = 8.3, 2.6 Hz, 1H), 3.87 (s, 3H); ^{13}C NMR (126 MHz, CD_2Cl_2): δ (ppm) = 194.3, 161.0, 144.0, 138.5, 137.3, 137.0, 132.1, 129.9, 129.0, 128.8, 128.7, 127.9, 125.4, 124.7, 120.0, 118.7, 110.6, 56.2; HRMS (EI): *m/z* (%) = 260.0836 (calcd for $C_{18}H_{12}O_2$: 260.0837); IR (KBr pellet): ν (cm^{-1}) = 2903, 1714, 1606, 1573, 1483, 1465, 1386, 1369, 1282, 1265, 1246, 1225, 1017, 1001, 868, 823, 774.

Copies of ^{1}H and ^{13}C spectra of all compounds

^{1}H NMR spectrum of 1-iodo-6,7-dimethoxyisoquinoline (8a)

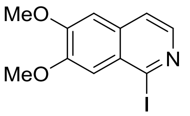

Frequency: 500 MHz

Solvent: $CDCl_3$

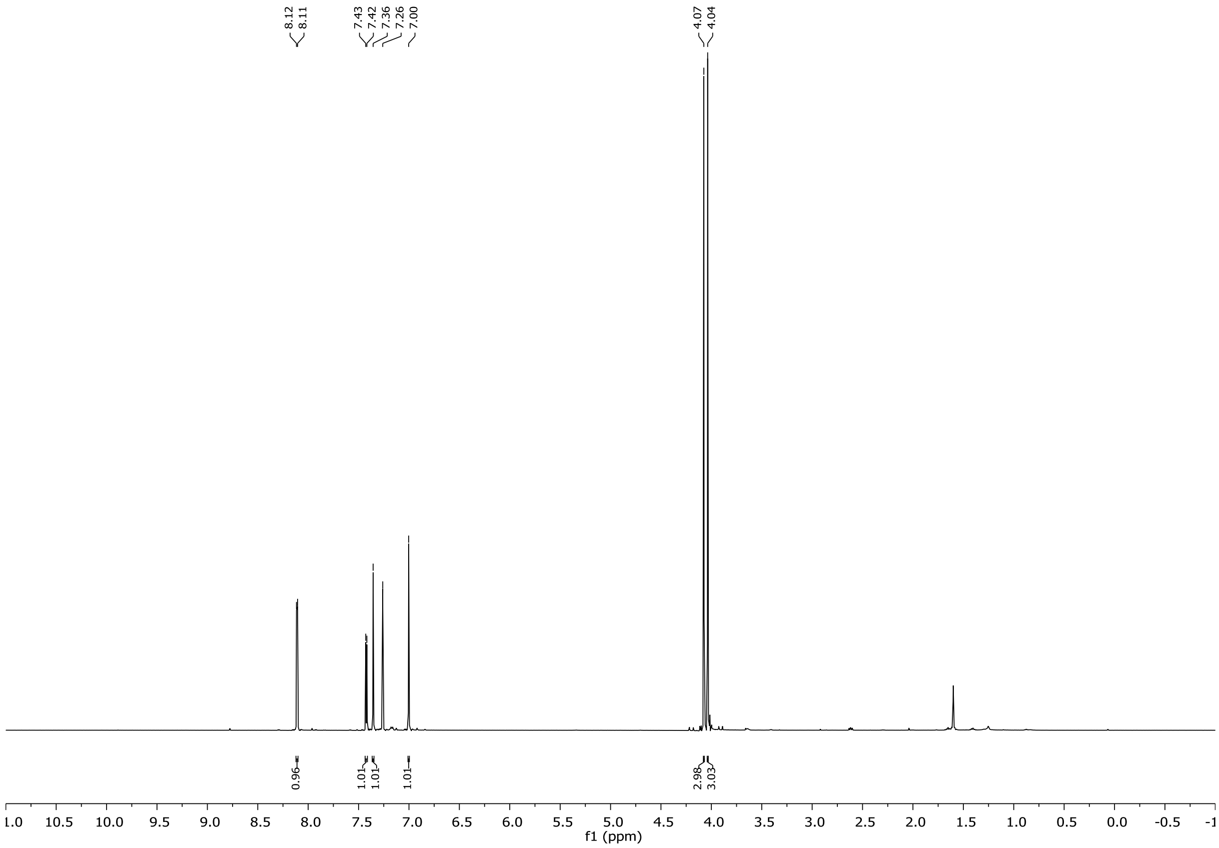

^{13}C NMR spectrum of 1-iodo-6,7-dimethoxyisoquinoline (8a)

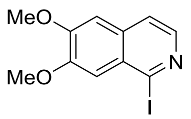

Frequency: 126 MHz

Solvent: $CDCl_3$

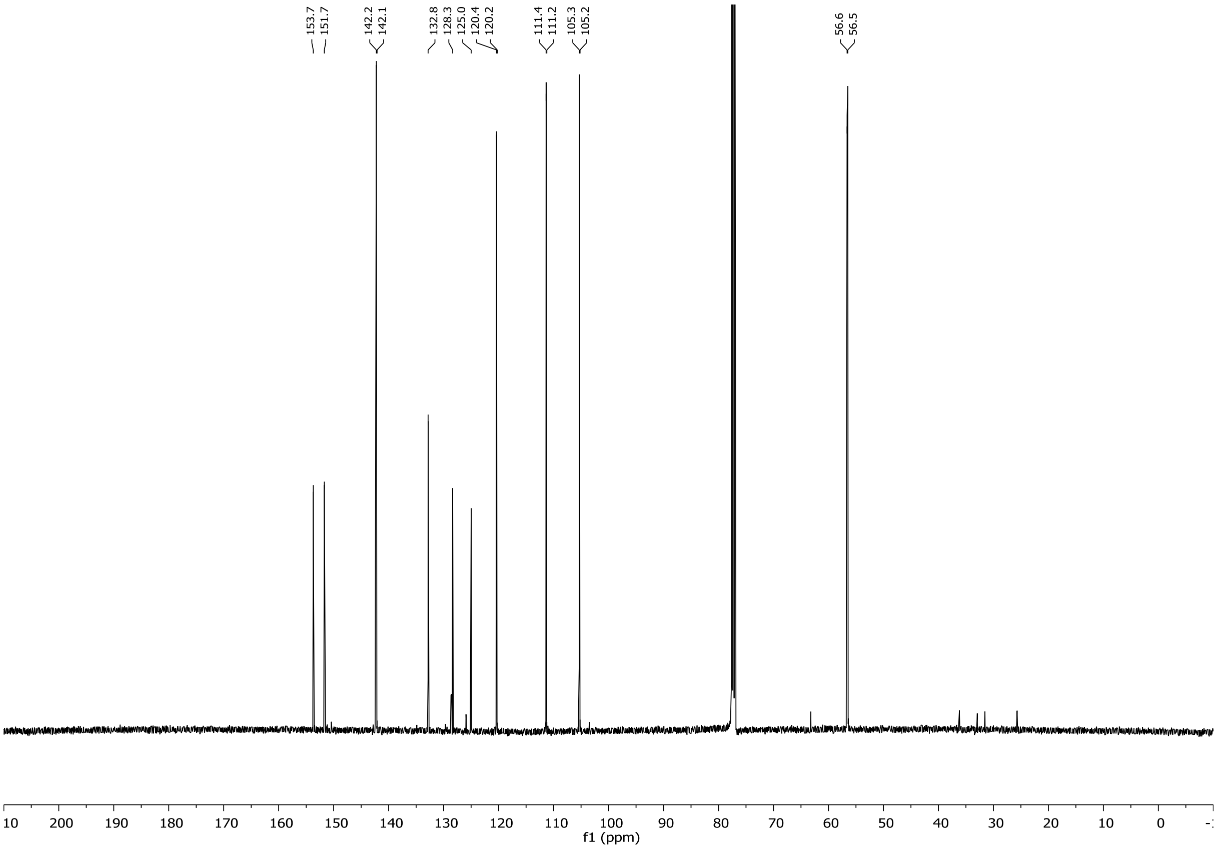

^{1}H NMR spectrum of 1-iodo-5,6,7-trimethoxyisoquinoline (8b)

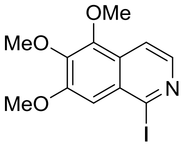

Frequency: 400 MHz

Solvent: $CDCl_3$

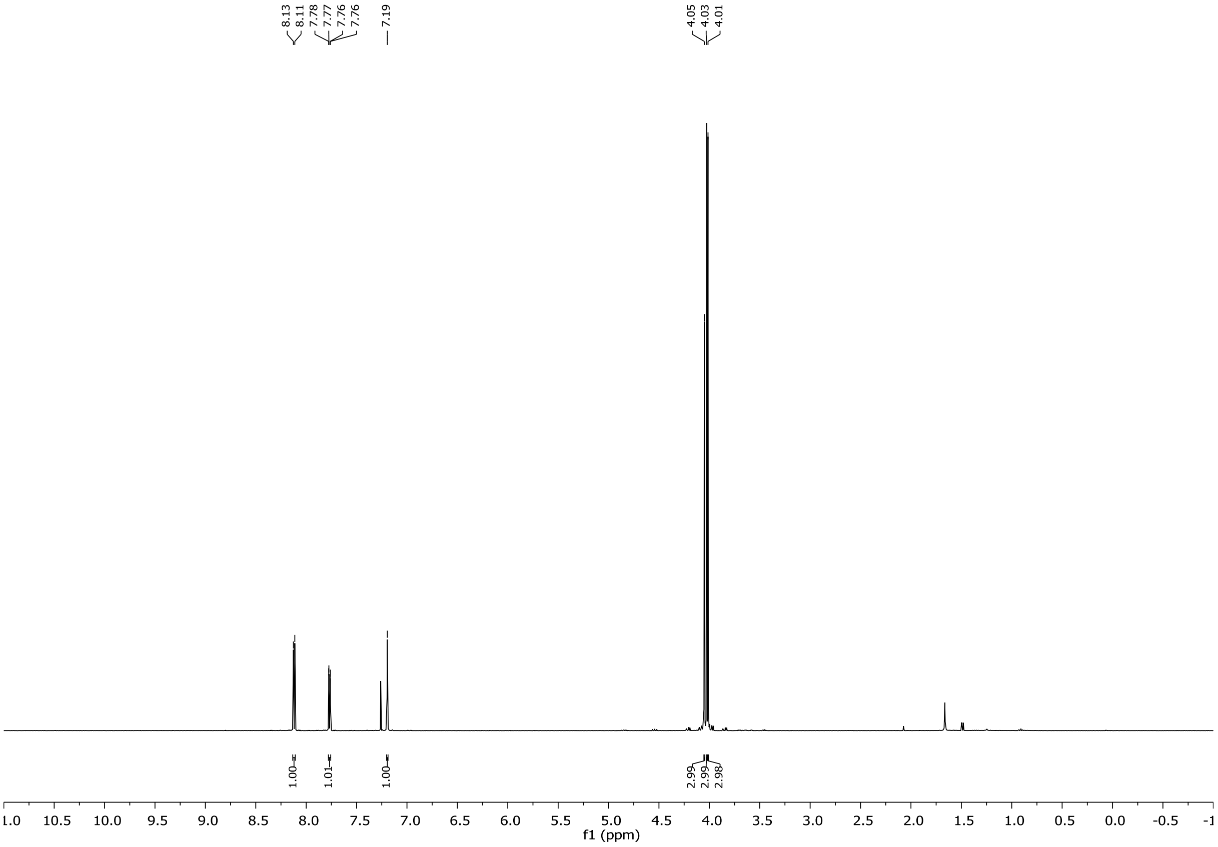

^{13}C NMR spectrum of 1-iodo-5,6,7-trimethoxyisoquinoline (8b)

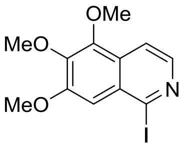

Frequency: 101 MHz

Solvent: $CDCl_3$

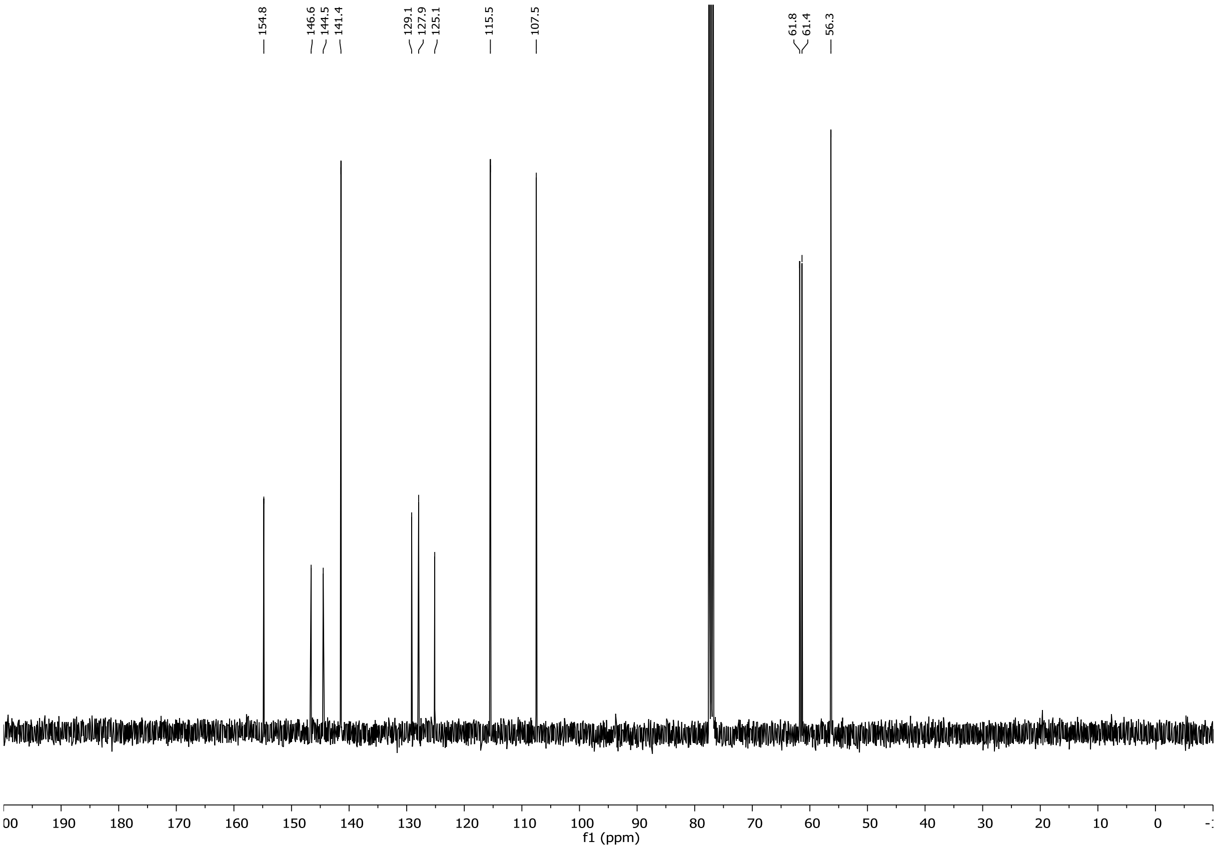

^{1}H NMR spectrum of 1-iodo-6-methoxyisoquinoline (8c)

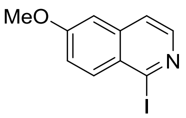

Frequency: 400 MHz

Solvent: $CDCl_3$

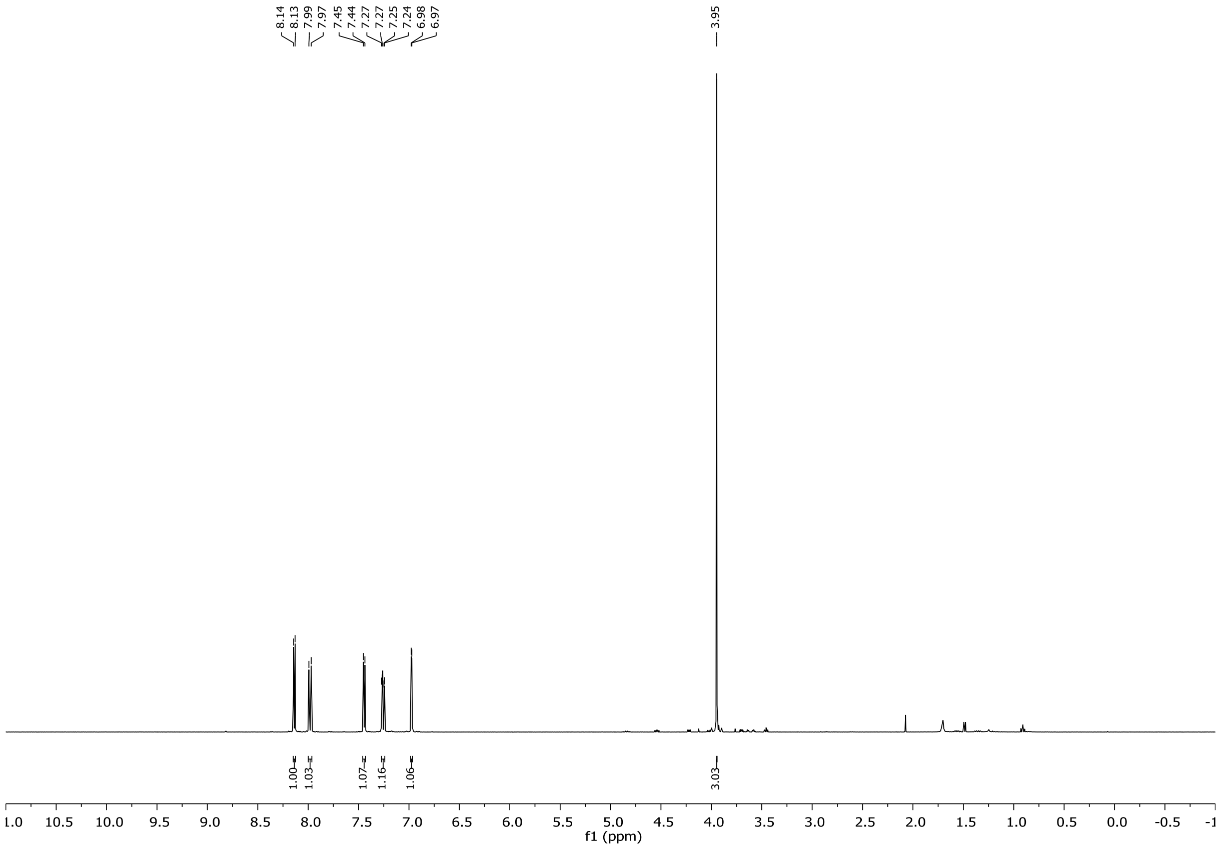

^{13}C NMR spectrum of 1-iodo-6-methoxyisoquinoline (8c)

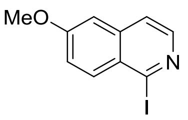

Frequency: 101 MHz

Solvent: $CDCl_3$

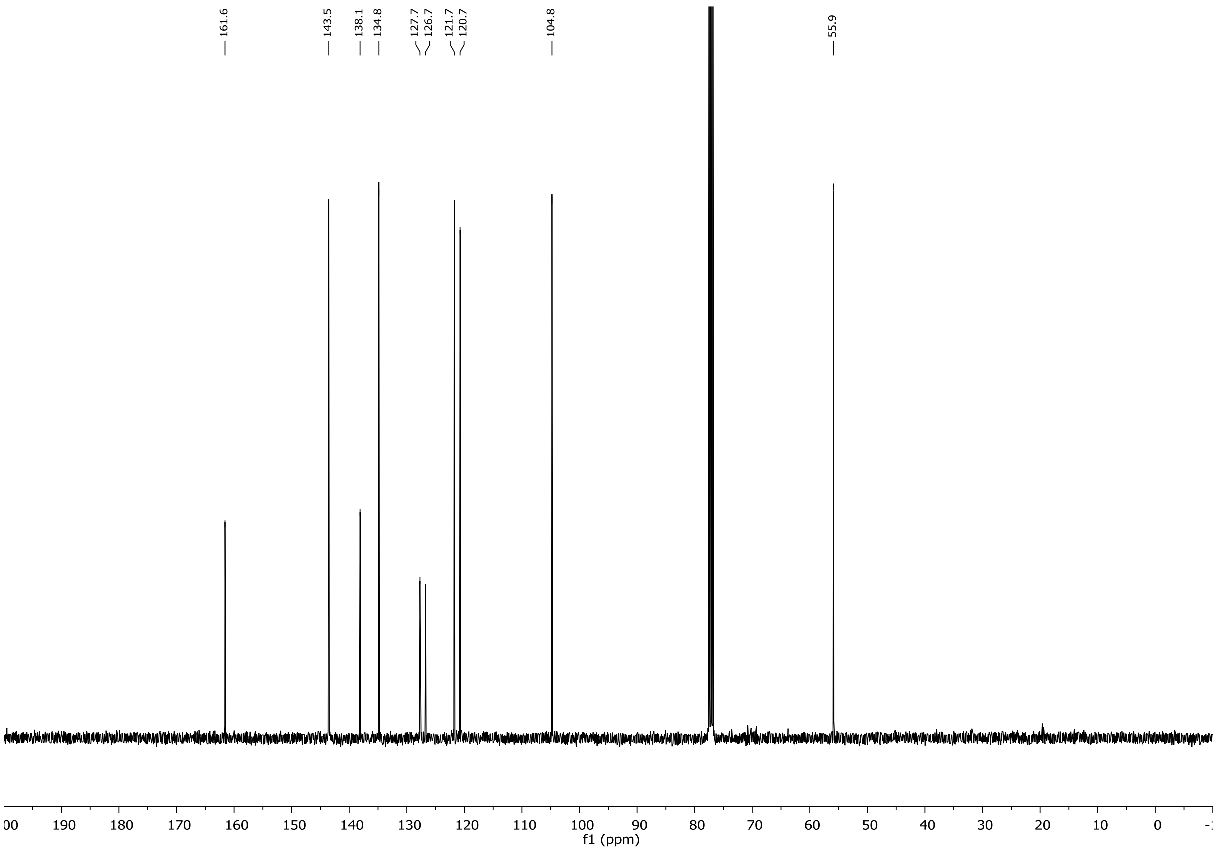

^{1}H NMR spectrum of methyl 2-(6,7-dimethoxyisoquinolin-1-yl)-5-methoxybenzoate (10a)

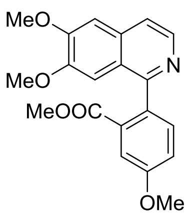

Frequency: 400 MHz

Solvent: CD_2Cl_2

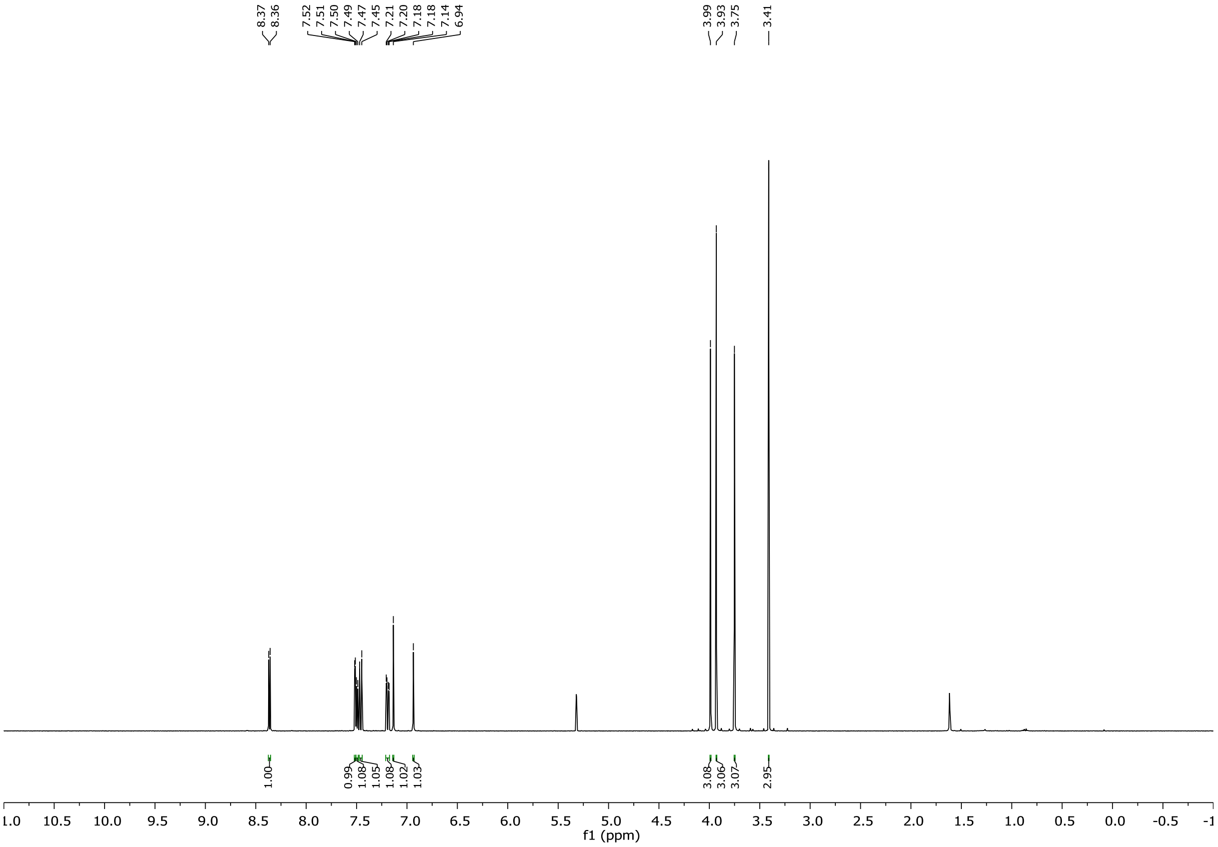

^{13}C NMR spectrum of methyl 2-(6,7-dimethoxyisoquinolin-1-yl)-5-methoxybenzoate (10a)

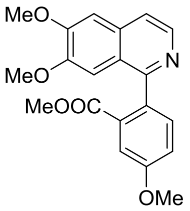

Frequency: 101 MHz

Solvent: CD_2Cl_2

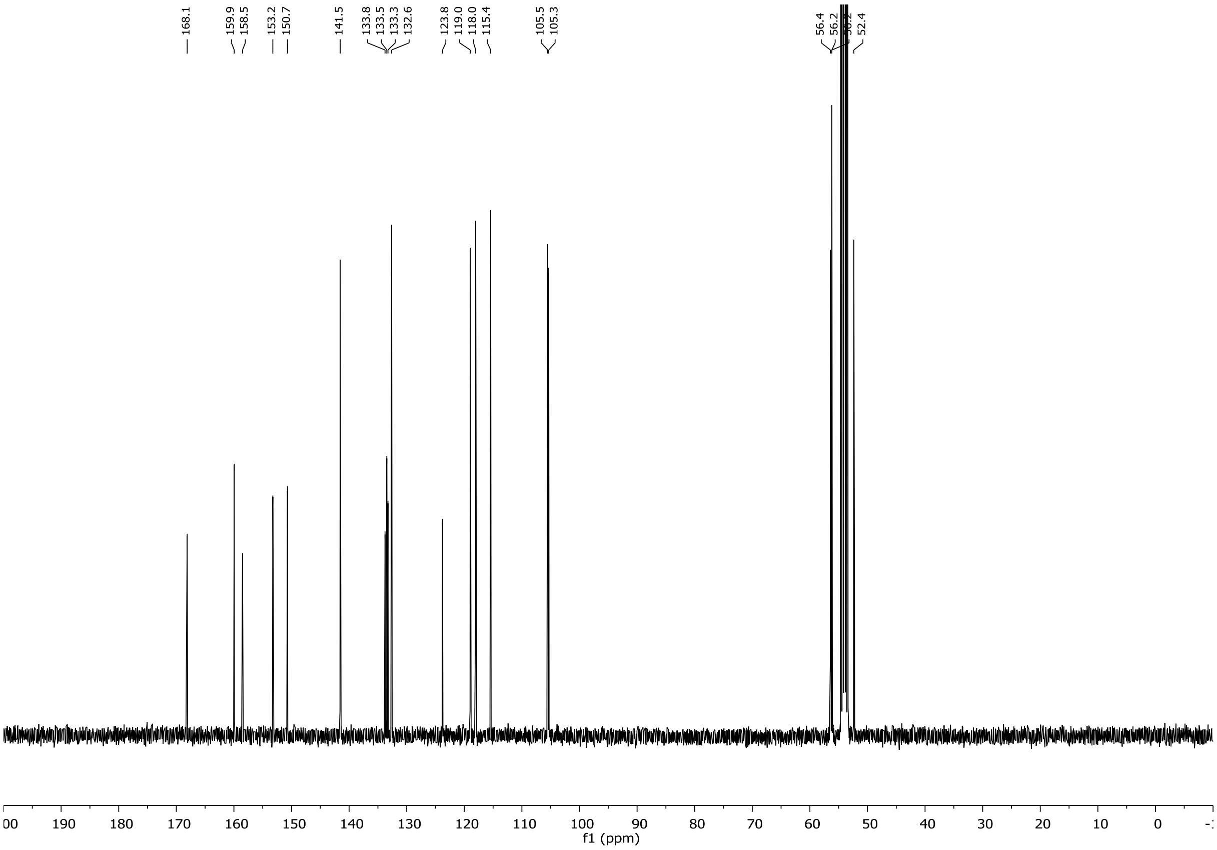

^{1}H NMR spectrum of methyl 2-(5,6,7-trimethoxyisoquinolin-1-yl)-5-methoxybenzoate (10b)

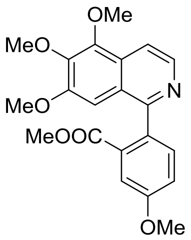

Frequency: 400 MHz

Solvent: CD_2Cl_2

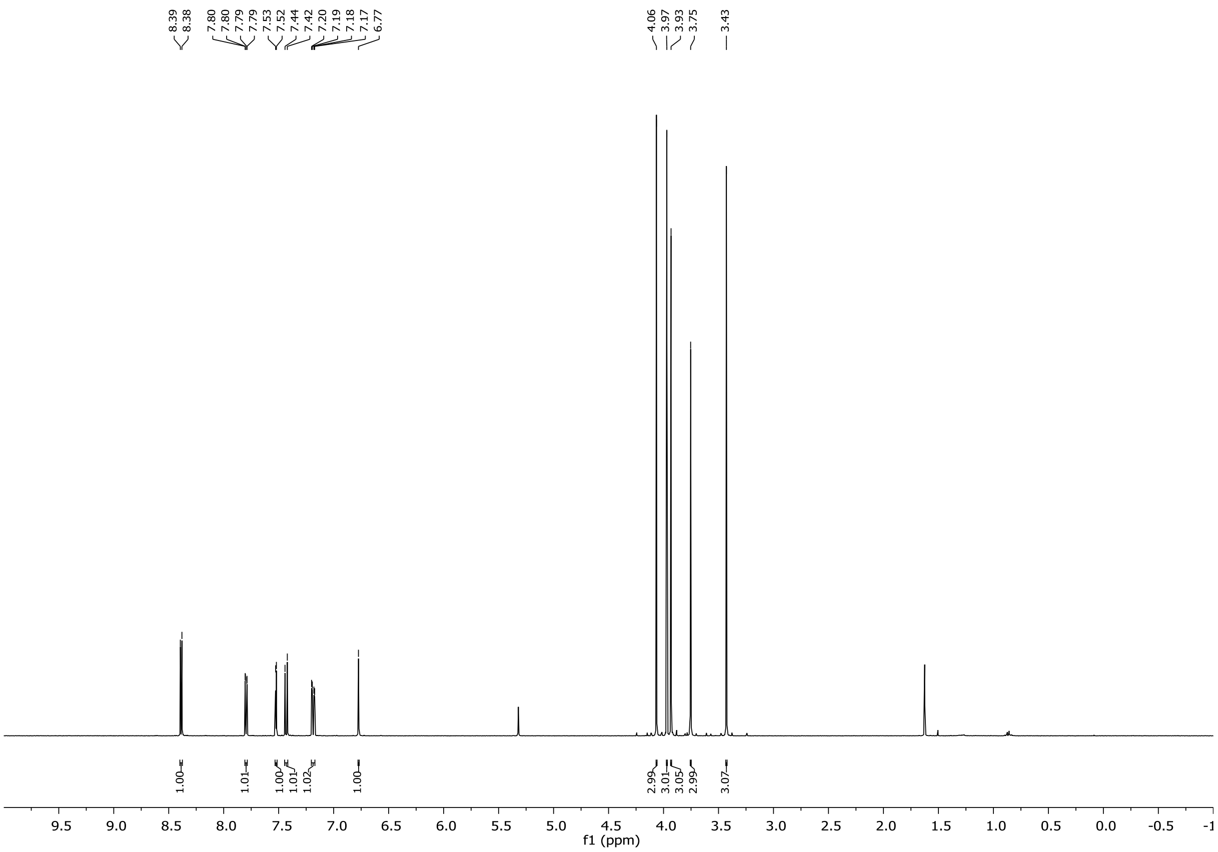

^{13}C NMR spectrum of methyl 2-(5,6,7-trimethoxyisoquinolin-1-yl)-5-methoxybenzoate (10b)

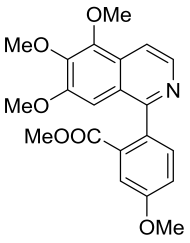

Frequency: 101 MHz

Solvent: CD_2Cl_2

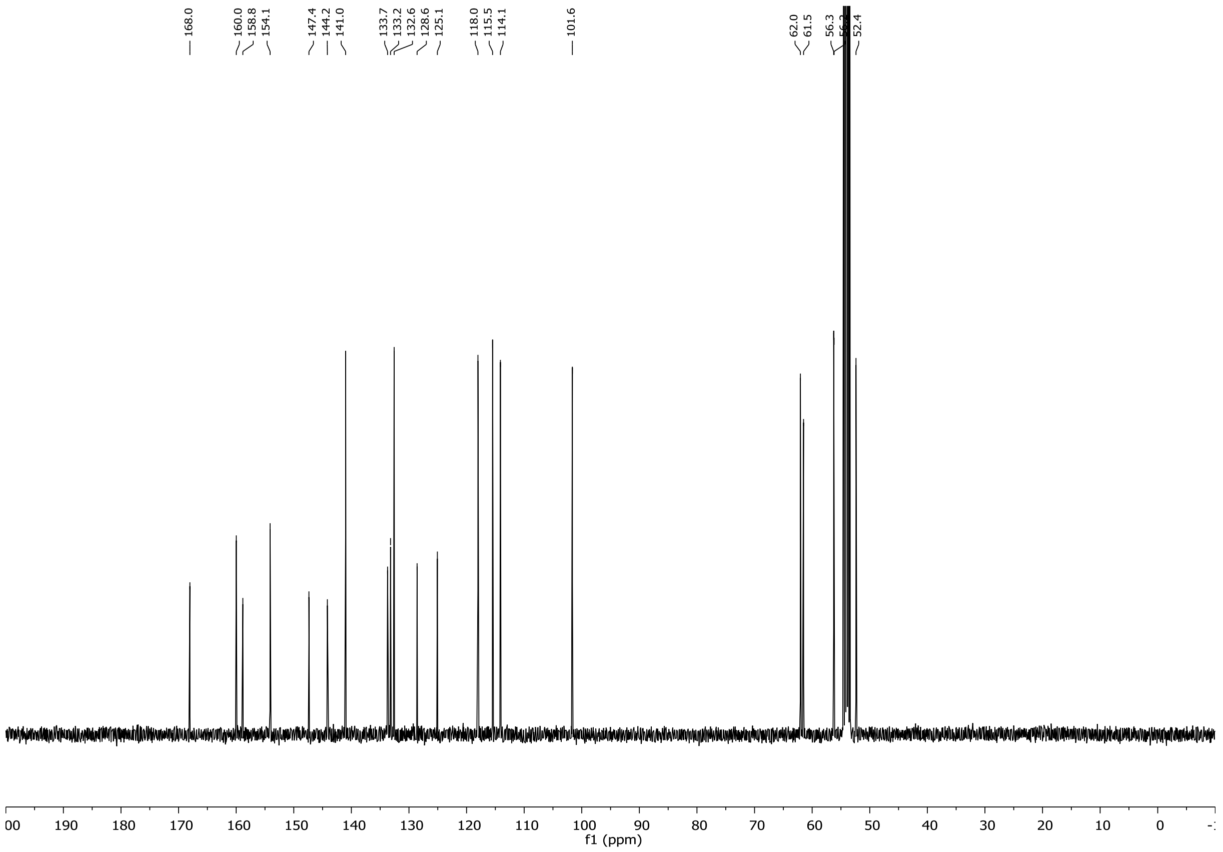

^{1}H NMR spectrum of methyl 2-(6-methoxyisoquinolin-1-yl)-5-methoxybenzoate (10c)

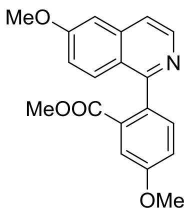

Frequency: 500 MHz

Solvent: $CDCl_3$

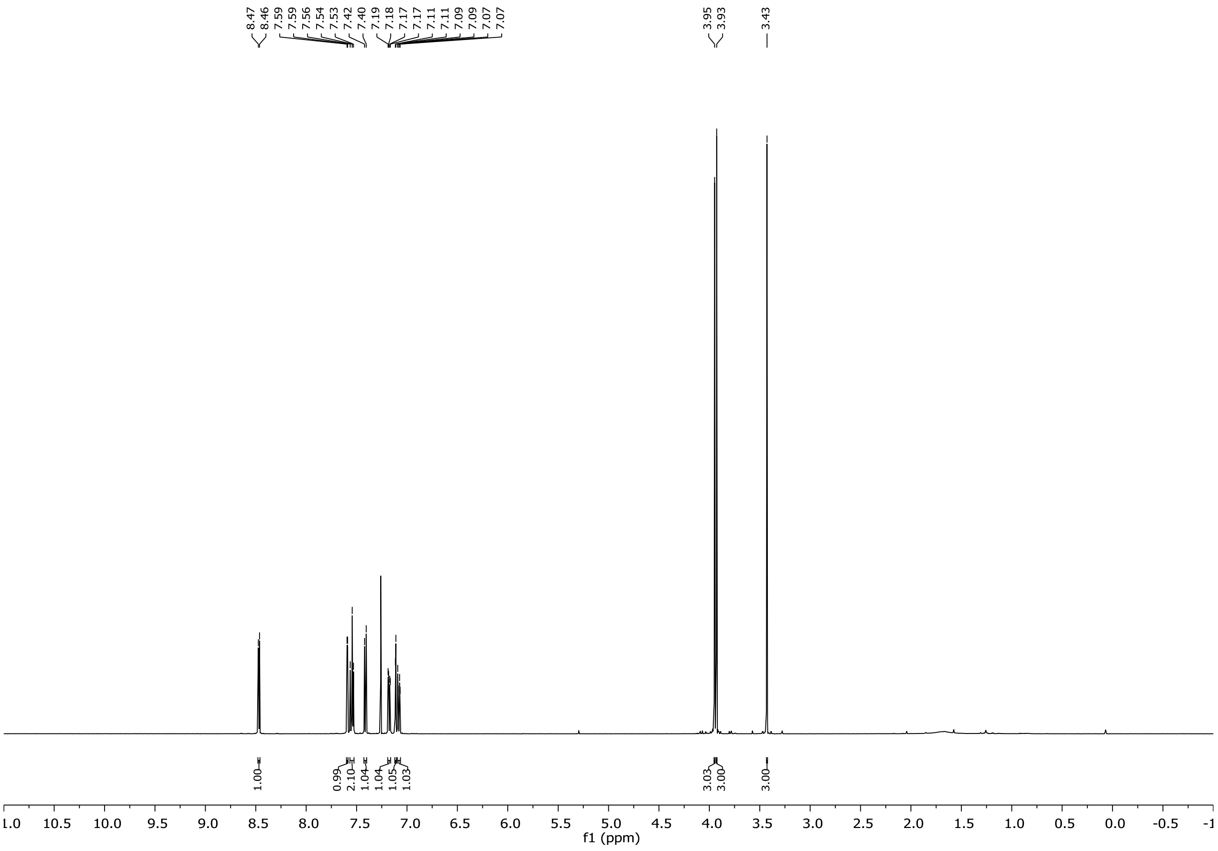

^{13}C NMR spectrum of methyl 2-(6-methoxyisoquinolin-1-yl)-5-methoxybenzoate (10c)

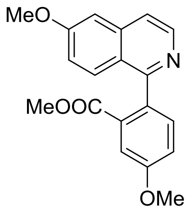

Frequency: 126 MHz

Solvent: $CDCl_3$

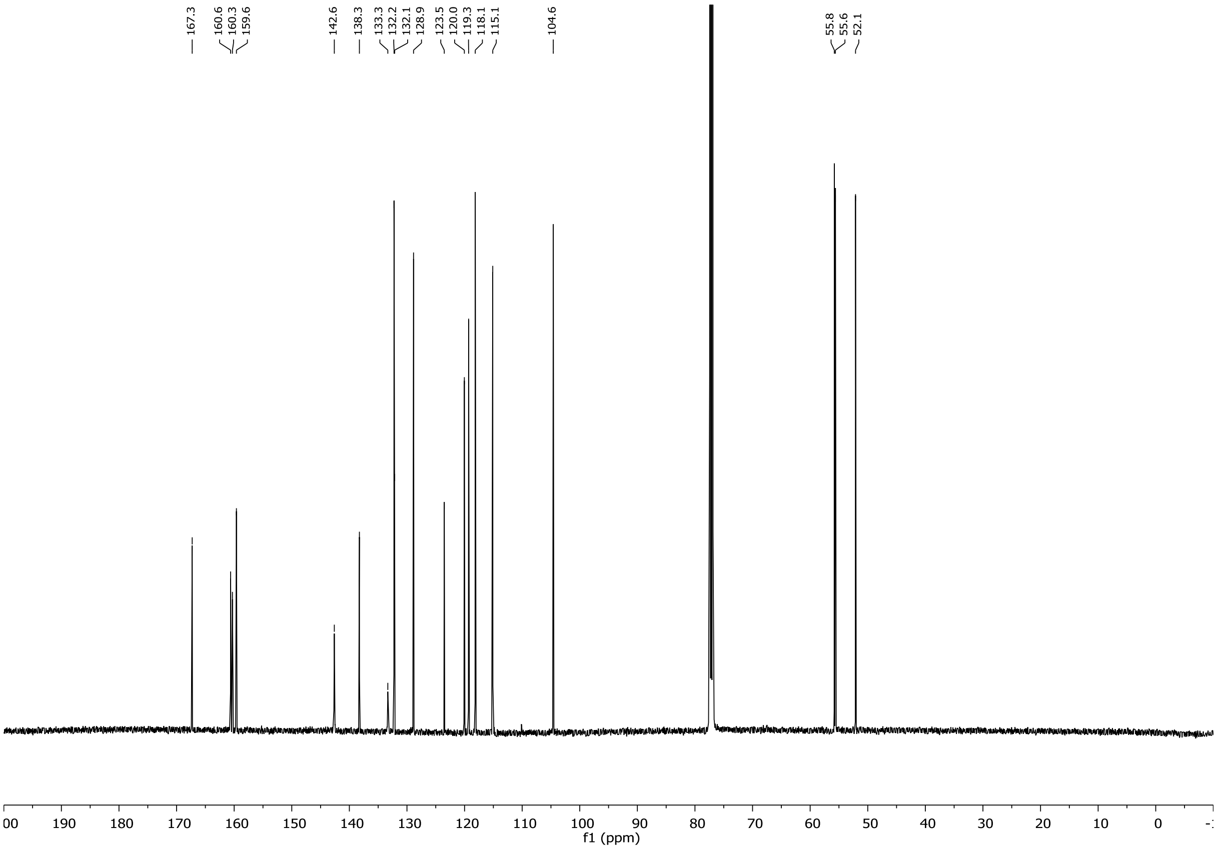

^{1}H NMR spectrum of 6-hydroxy-5,9-dimethoxy-7*H*-dibenzo[*de,h*]quinolin-7-one (6-*O*-demethylmenisporphine, 4)

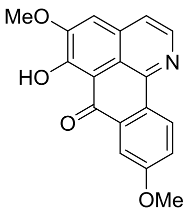

Frequency: 500 MHz

Solvent: $CDCl_3$

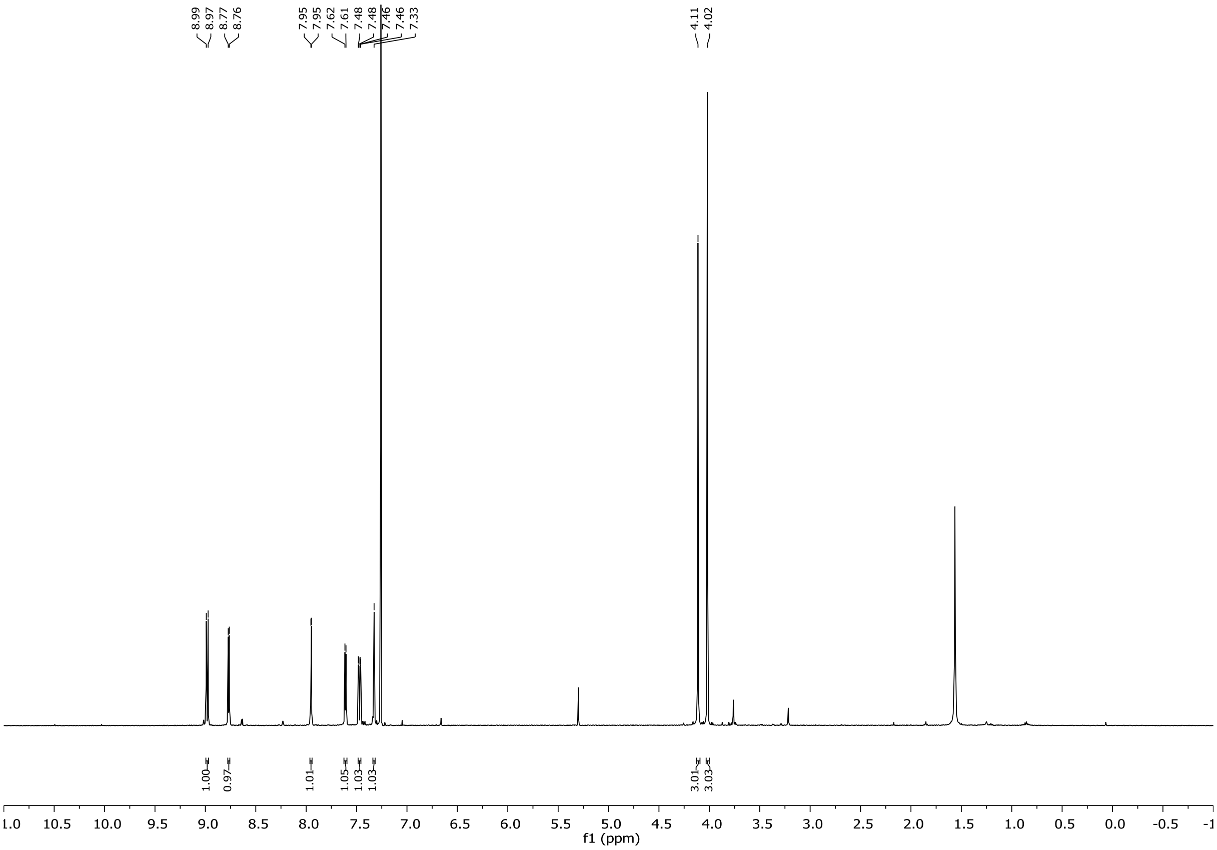

^{13}C NMR spectrum of 6-hydroxy-5,9-dimethoxy-7*H*-dibenzo[*de,h*]quinolin-7-one (6-*O*-demethylmenisporphine, 4)

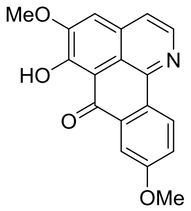

Frequency: 126 MHz

Solvent: $CDCl_3$

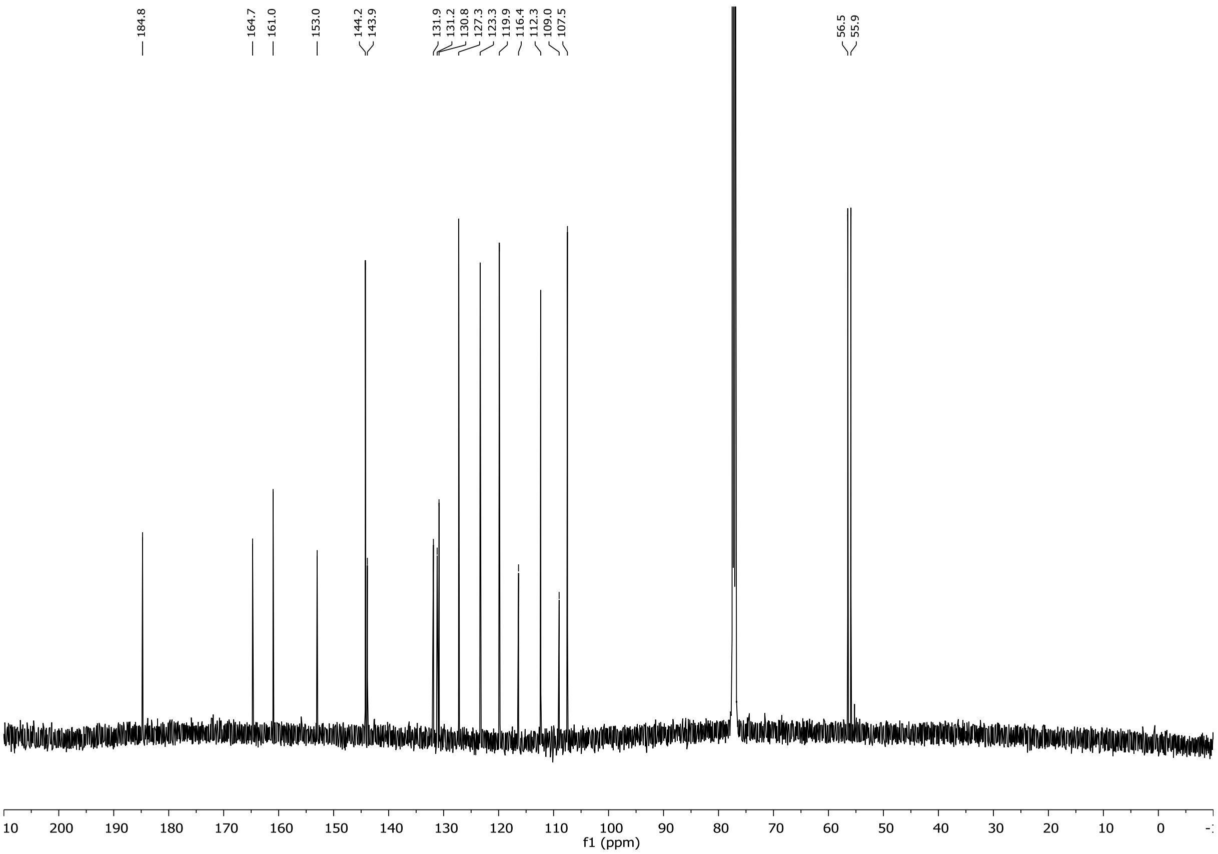

^{1}H NMR spectrum of 6-hydroxy-4,5,9-trimethoxy-7*H*-dibenzo[*de,h*]quinolin-7-one (dauriporphinoline, 5)

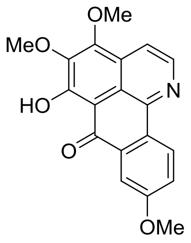

Frequency: 500 MHz

Solvent: $CDCl_3$

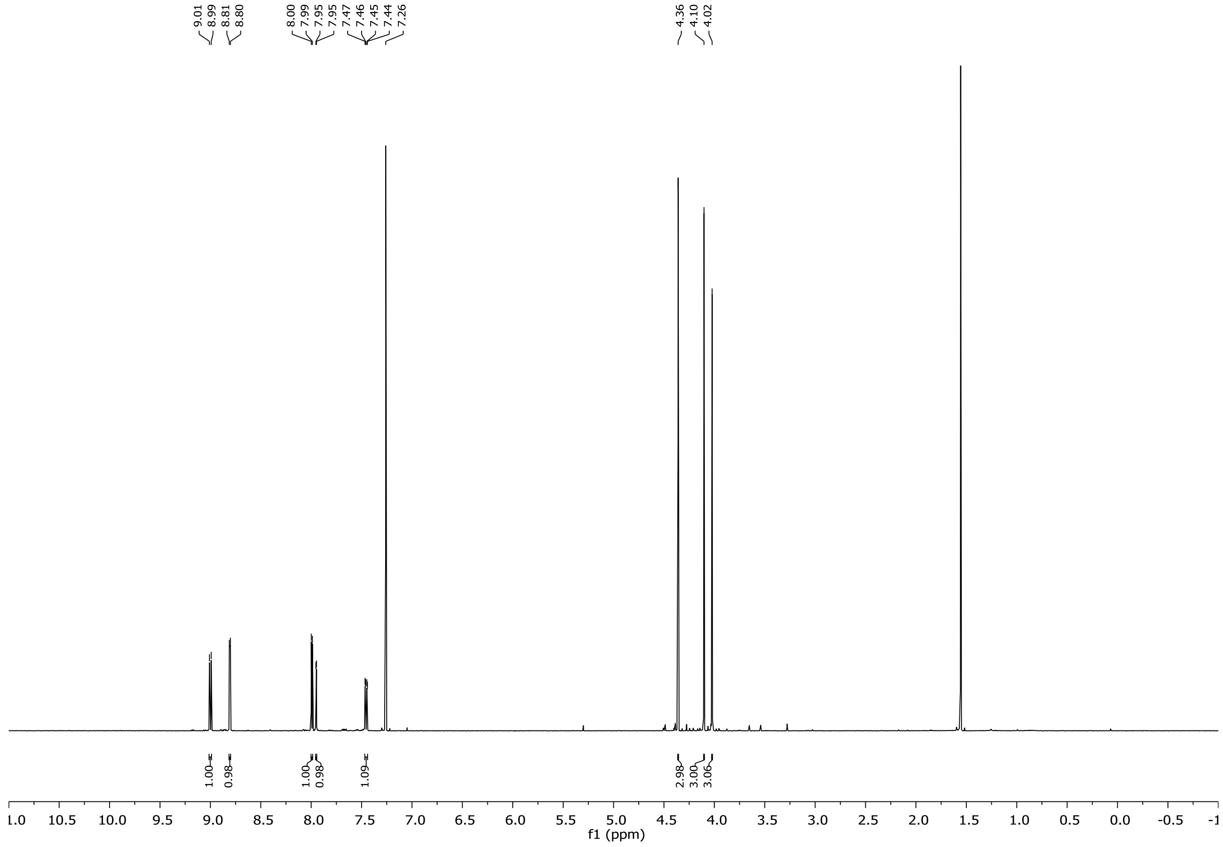

^{13}C NMR spectrum of 6-hydroxy-4,5,9-trimethoxy-7*H*-dibenzo[*de,h*]quinolin-7-one (dauriporphinoline, 5)

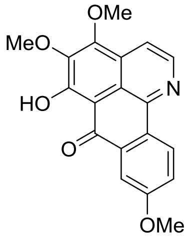

Frequency: 126 MHz

Solvent: $CDCl_3$

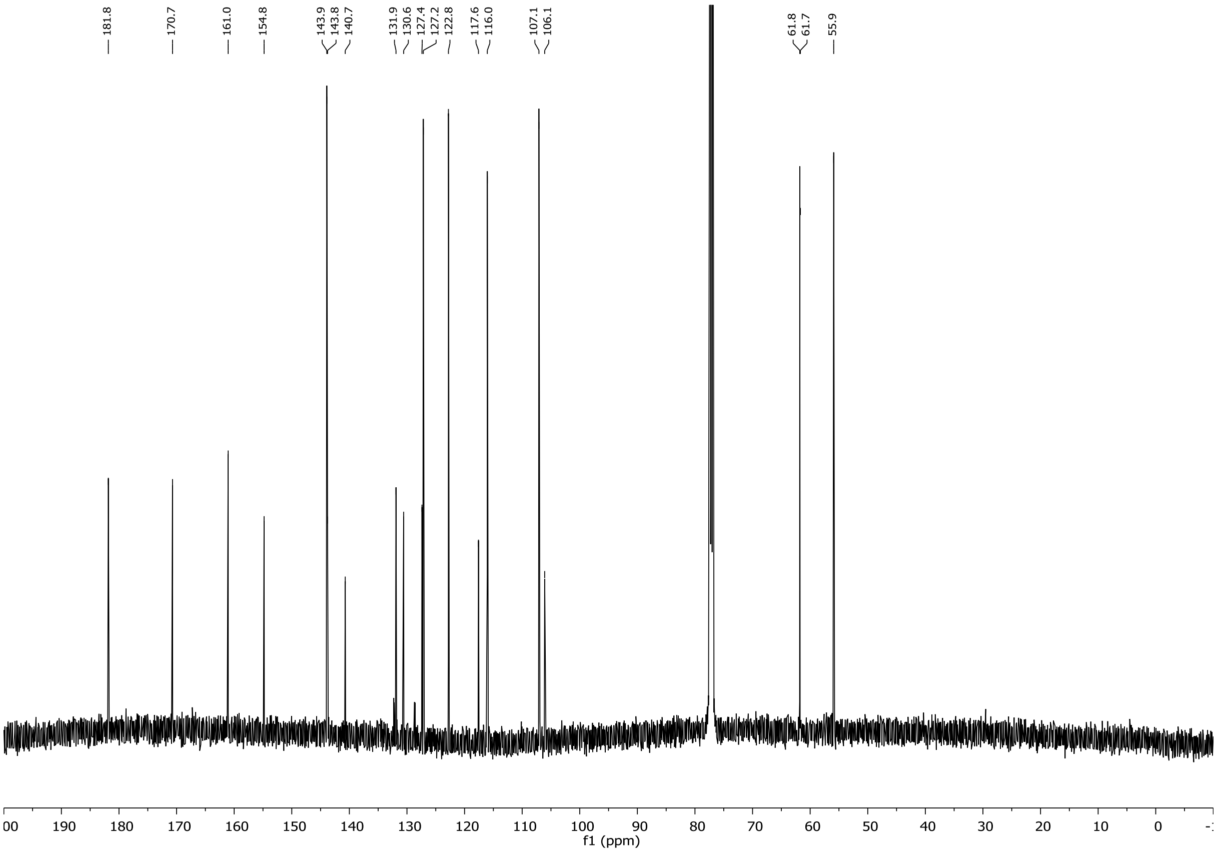

^{1}H NMR spectrum of 5,9-dimethoxy-7*H*-dibenzo[*de,h*]quinolin-7-one (bianfugecine, 6)

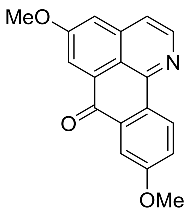

Frequency: 400 MHz

Solvent: $CDCl_3$

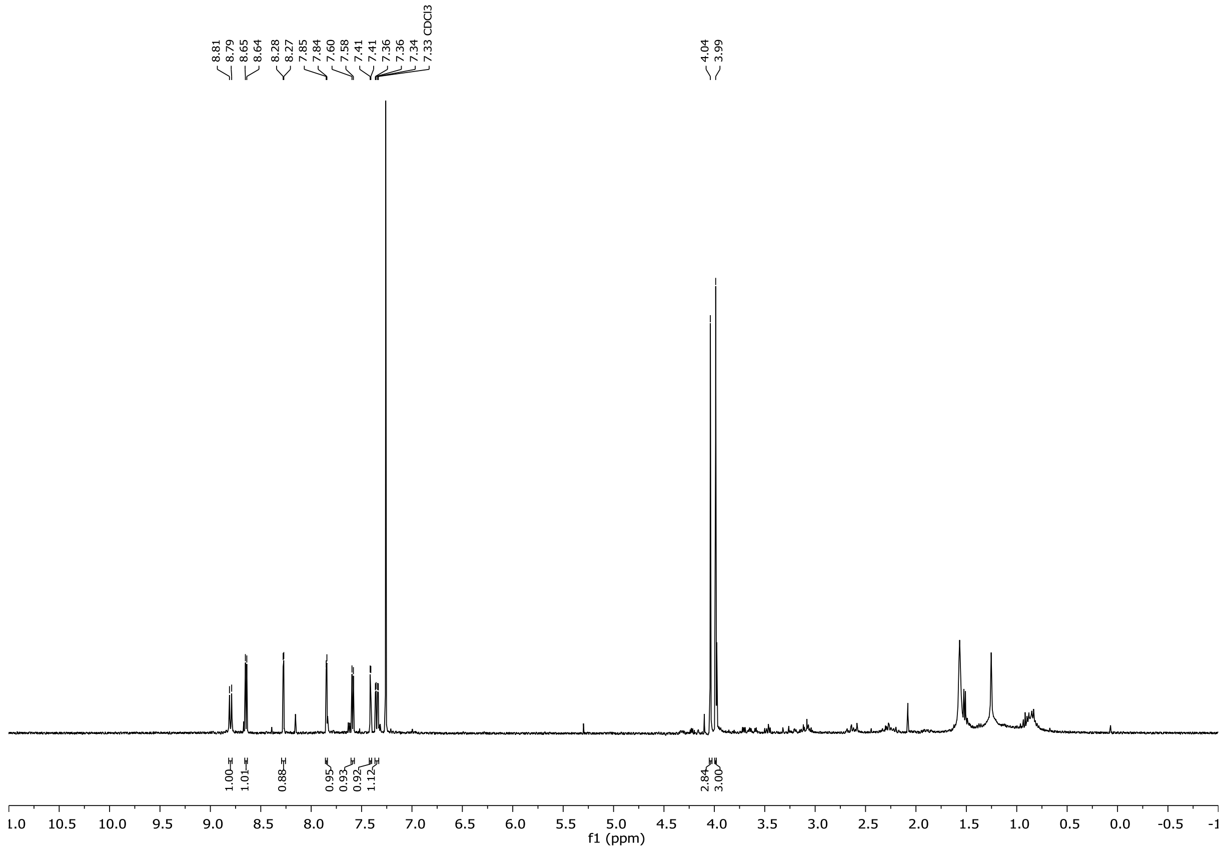

^{13}C NMR spectrum of 5,9-dimethoxy-7*H*-dibenzo[*de,h*]quinolin-7-one (bianfugecine, 6)

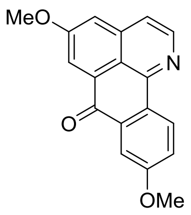

Frequency: 101 MHz

Solvent: $CDCl_3$

3 carbon resonance signals are not distinguishable from background noise

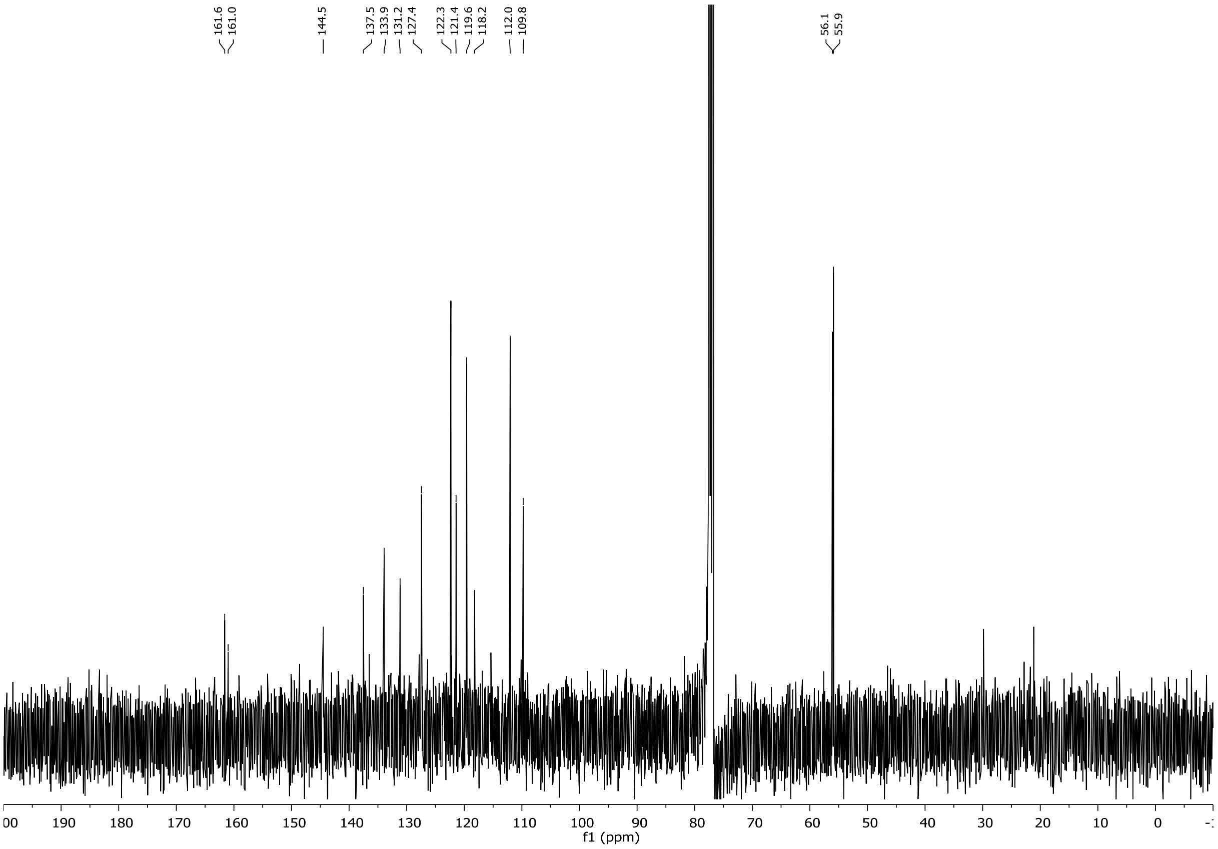

^{1}H NMR spectrum of 5,6,9-trimethoxy-7*H*-dibenzo[*de,h*]quinolin-7-one (menisporphine, 2)

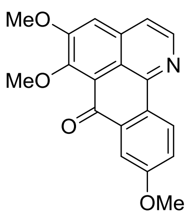

Frequency: 400 MHz

Solvent: $CDCl_3$

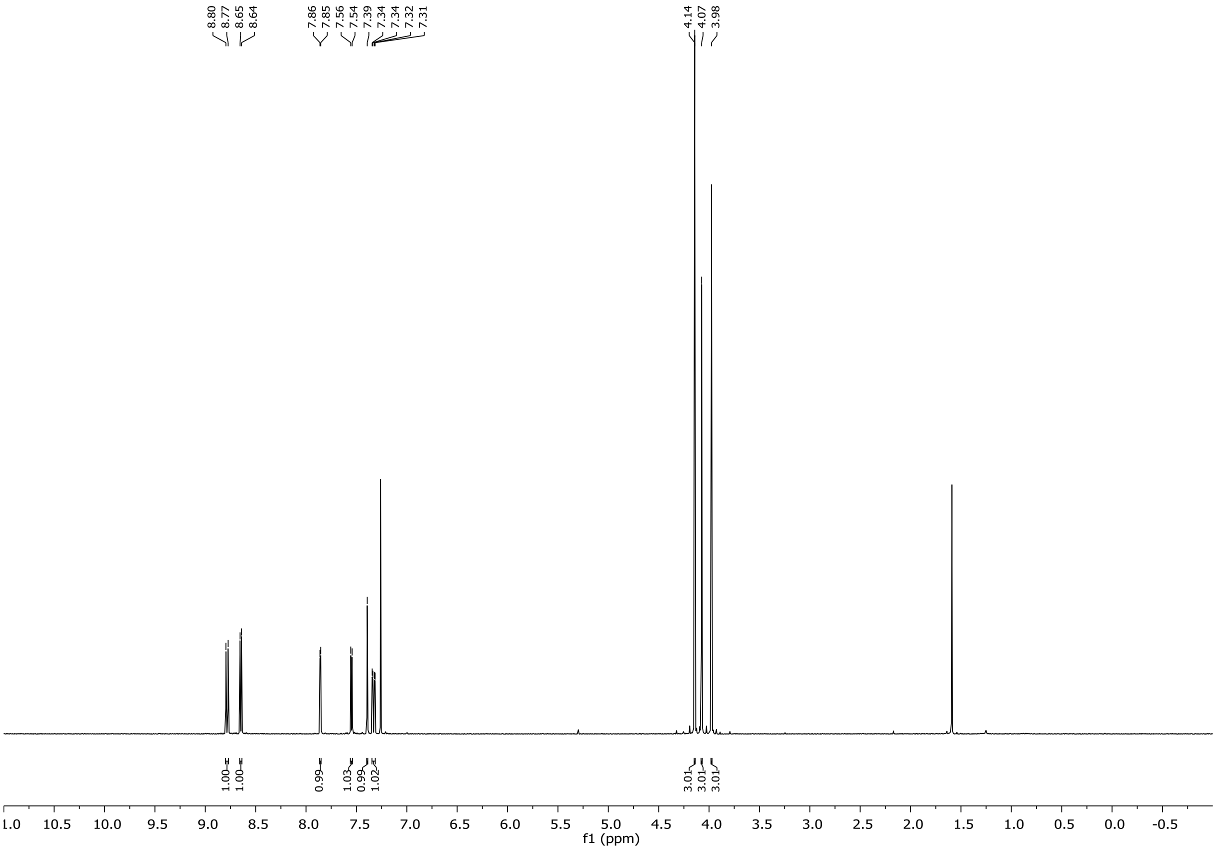

^{13}C NMR spectrum of 5,6,9-trimethoxy-7*H*-dibenzo[*de,h*]quinolin-7-one (menisporphine, 2)

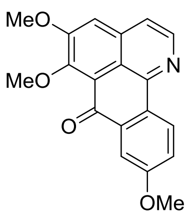

Frequency: 101 MHz

Solvent: $CDCl_3$

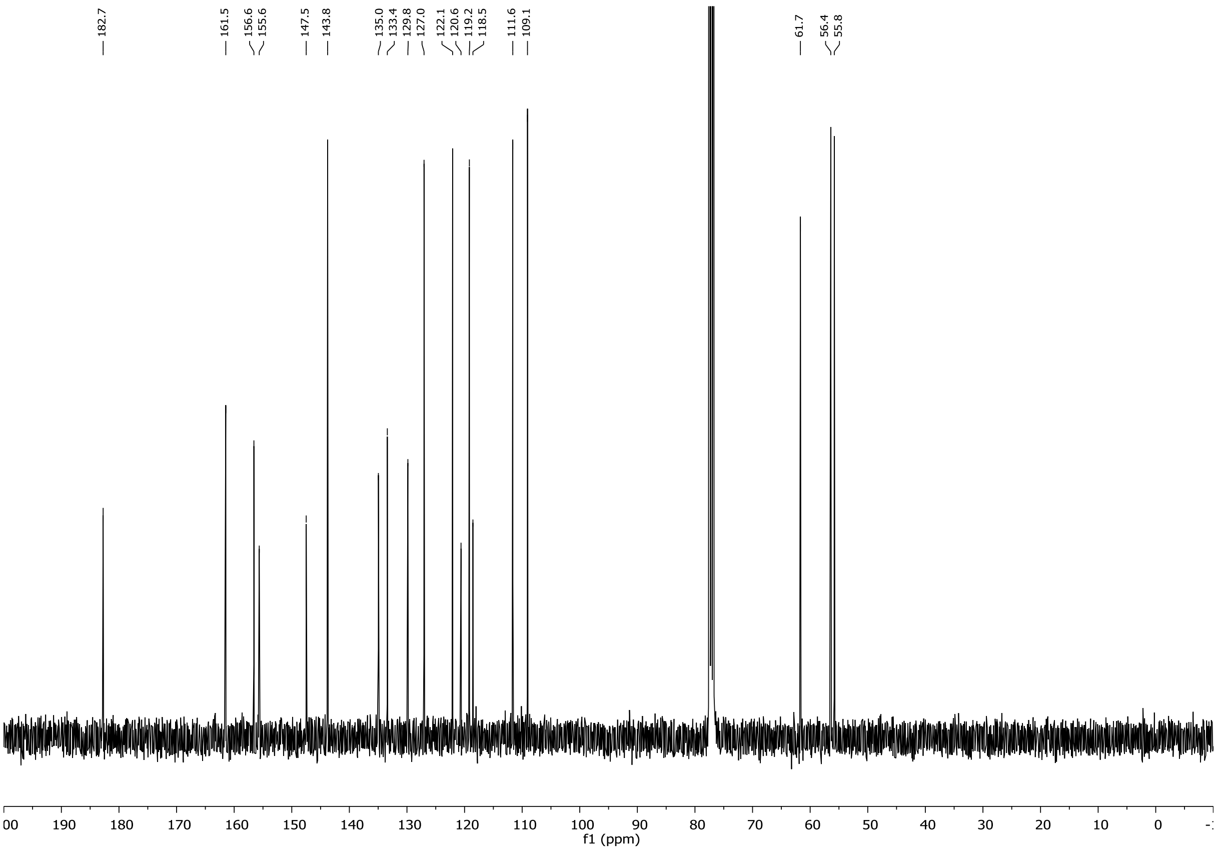

^{1}H NMR spectrum of 5,7,9-trimethoxy-6*H*-dibenzo[*de,h*]quinolin-6-one (18)

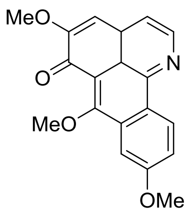

Frequency: 500 MHz

Solvent: $CDCl_3$

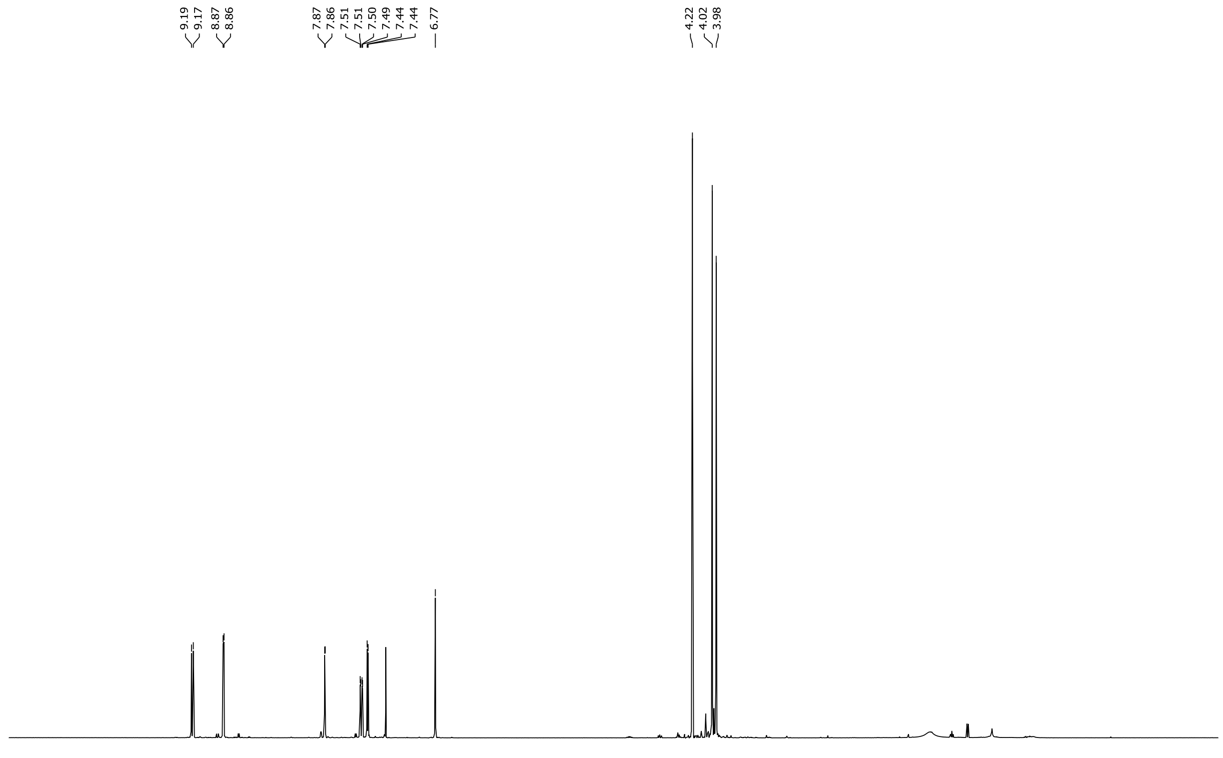

^{13}C NMR spectrum of 5,7,9-trimethoxy-6*H*-dibenzo[*de,h*]quinolin-6-one (18)

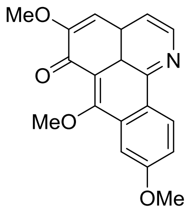

Frequency: 126 MHz

Solvent: $CDCl_3$

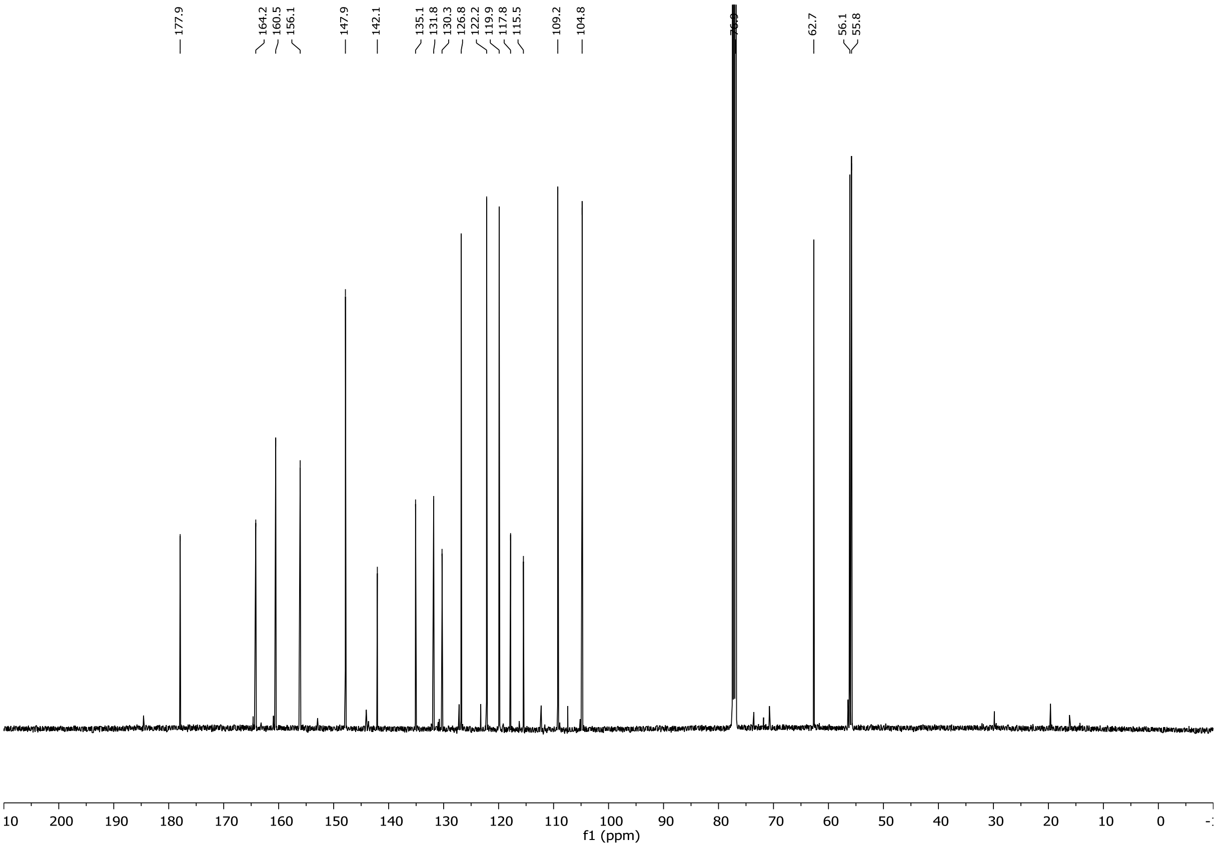

^{1}H NMR spectrum of 4,5,6,9-tetramethoxy-7*H*-dibenzo[*de,h*]quinolin-7-one (dauriporphine, 3)

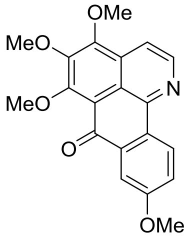

Frequency: 500 MHz

Solvent: $CDCl_3$

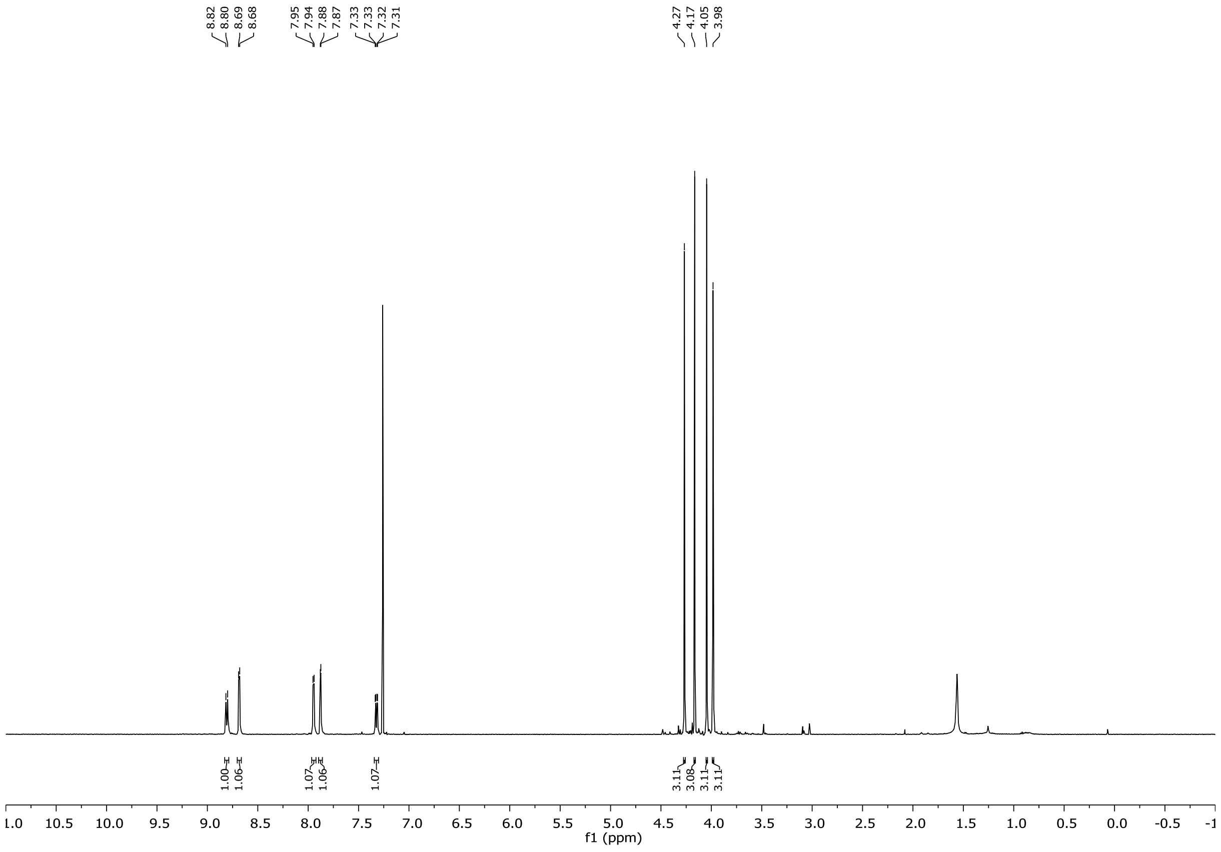

^{13}C NMR spectrum of 4,5,6,9-tetramethoxy-7*H*-dibenzo[*de,h*]quinolin-7-one (dauriporphine, 3)

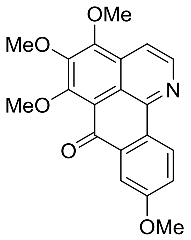

Frequency: 126 MHz

Solvent: $CDCl_3$

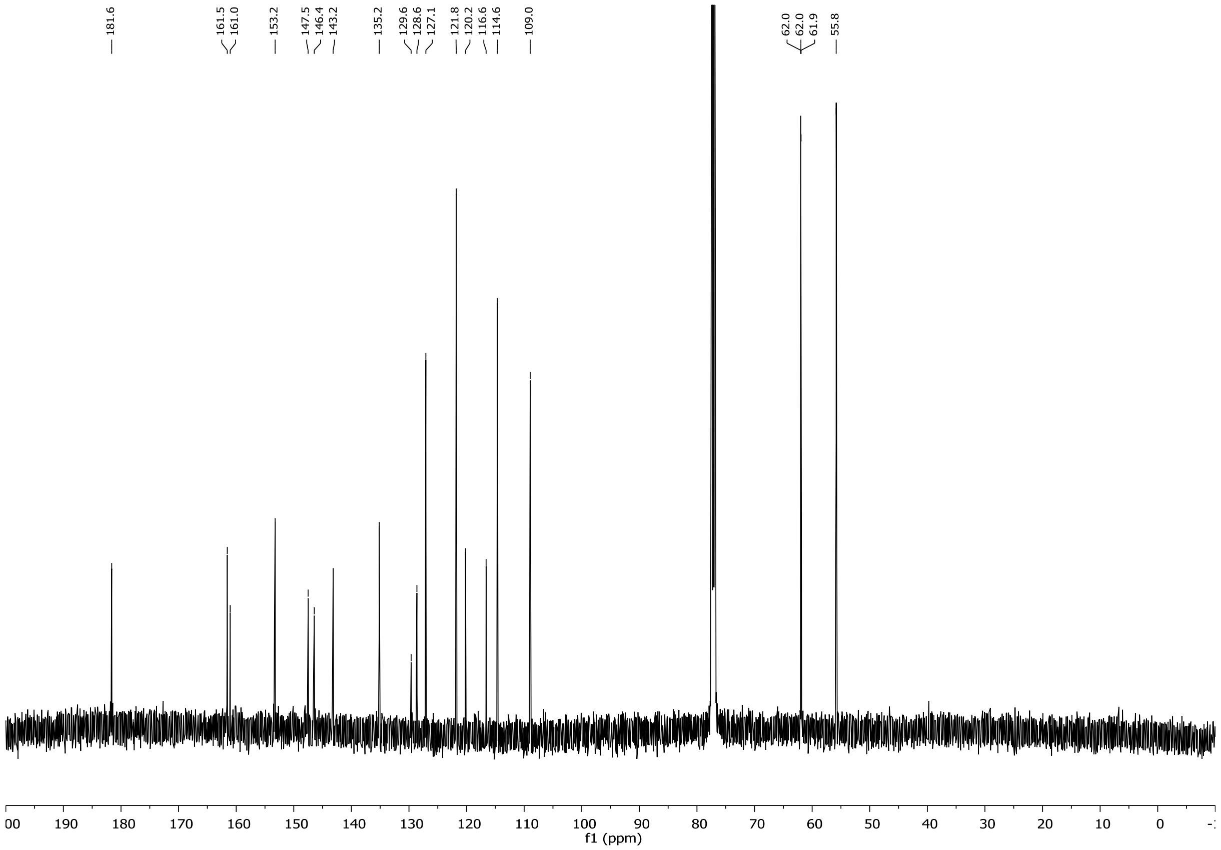

^{1}H NMR spectrum of 4,5,7,9-tetramethoxy-6*H*-dibenzo[*de,h*]quinolin-6-one (19)

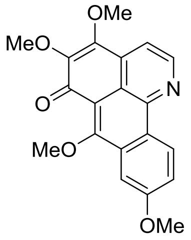

Frequency: 500 MHz

Solvent: $CDCl_3$

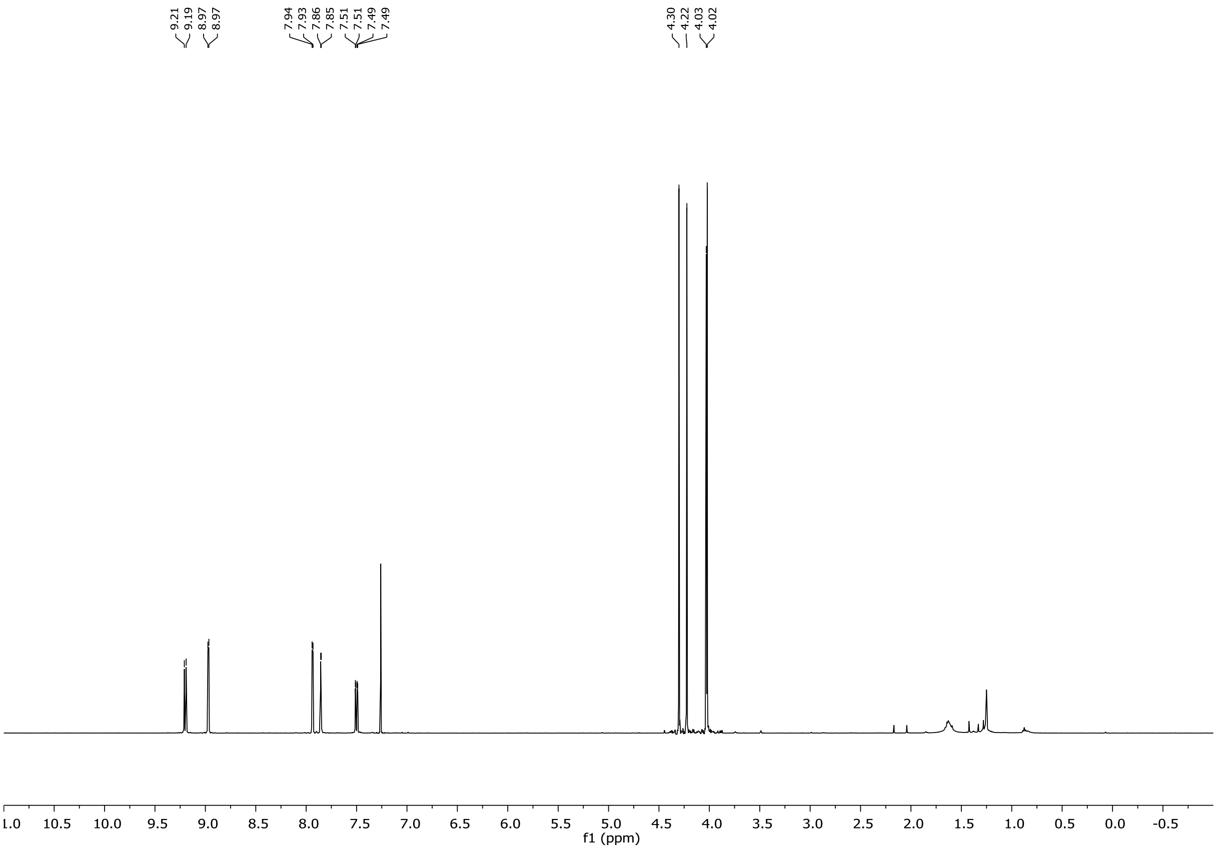

^{13}C NMR spectrum of 4,5,7,9-tetramethoxy-6*H*-dibenzo[*de,h*]quinolin-6-one (19)

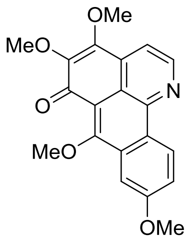

Frequency: 126 MHz

Solvent: $CDCl_3$

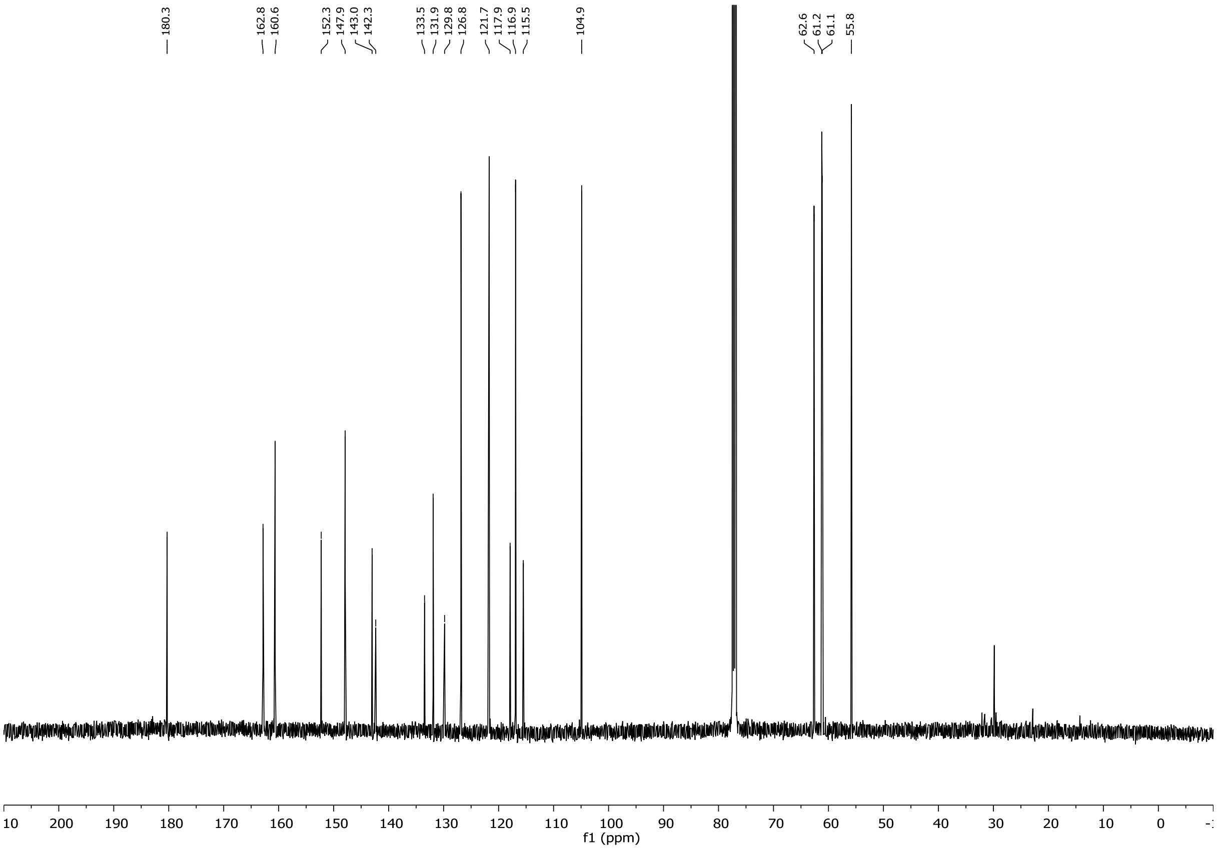

^{1}H NMR spectrum of *N*,*N*-diethyl-5-methoxy-2-(6-methoxyisoquinolin-1-yl)benzamide (12)

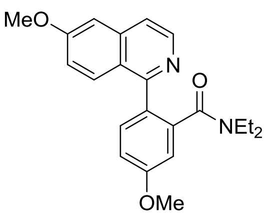

Frequency: 500 MHz

Solvent: CD_2Cl_2

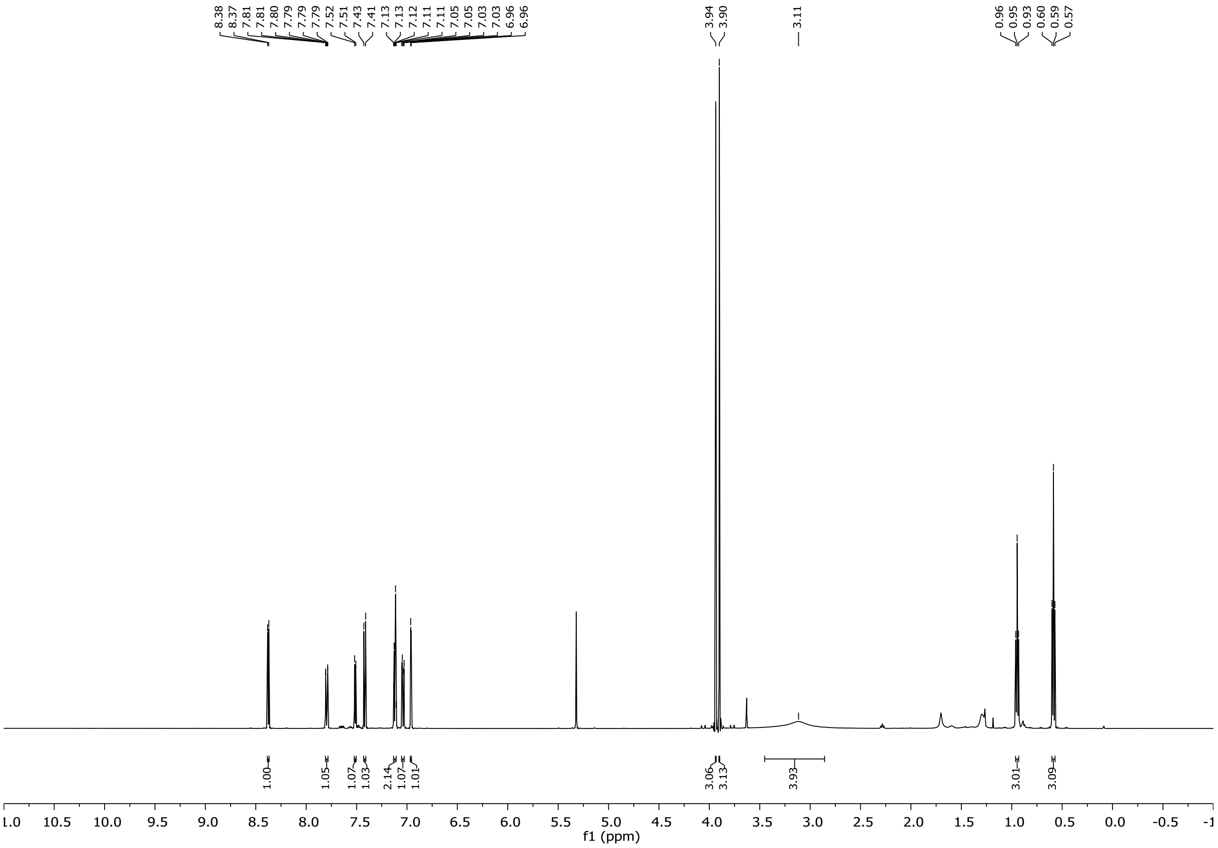

^{13}C NMR spectrum of *N*,*N*-diethyl-5-methoxy-2-(6-methoxyisoquinolin-1-yl)benzamide (12)

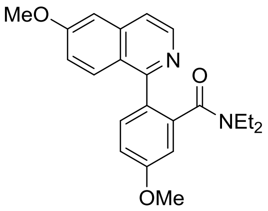

Frequency: 126 MHz

Solvent: CD_2Cl_2

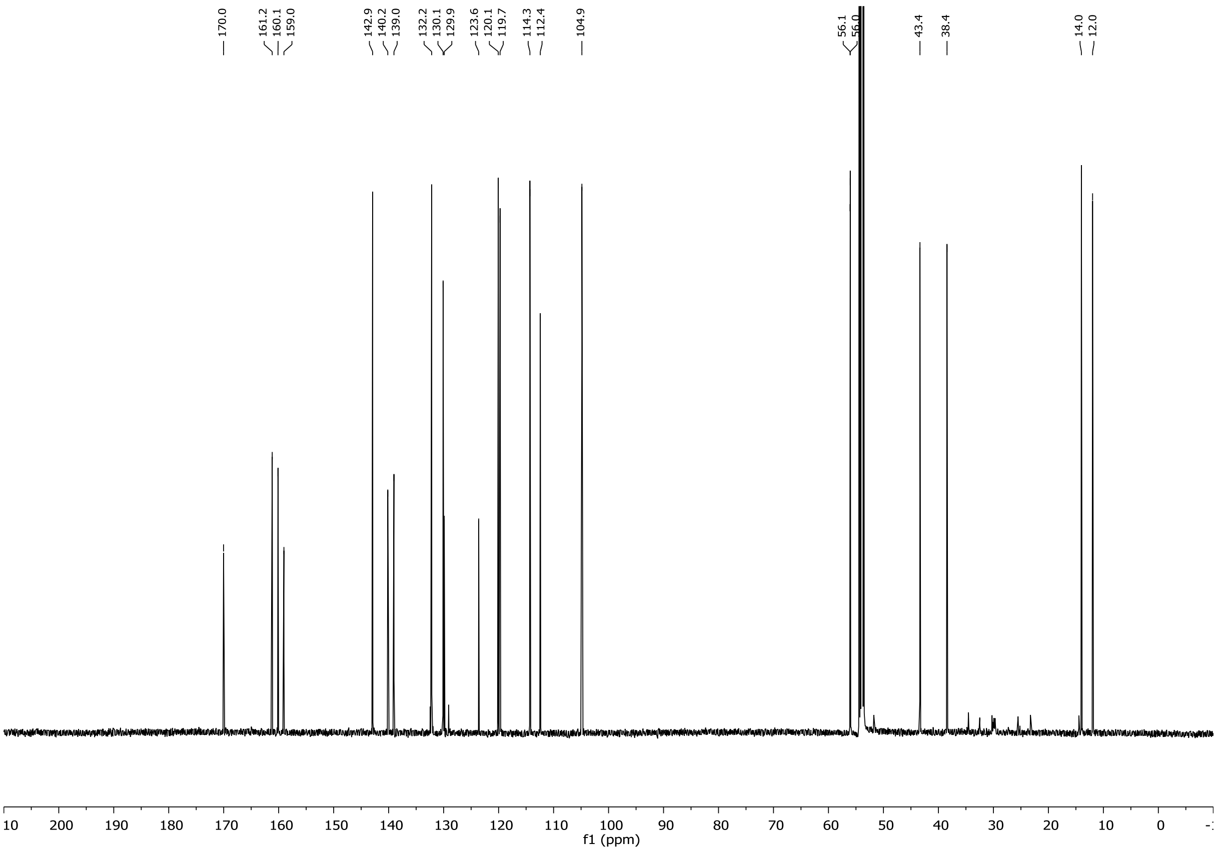

^{1}H NMR spectrum of methyl 5-methoxy-2-(naphthalen-1-yl)benzoate (15)

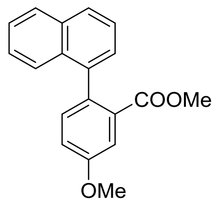

Frequency: 400 MHz

Solvent: CD_2Cl_2

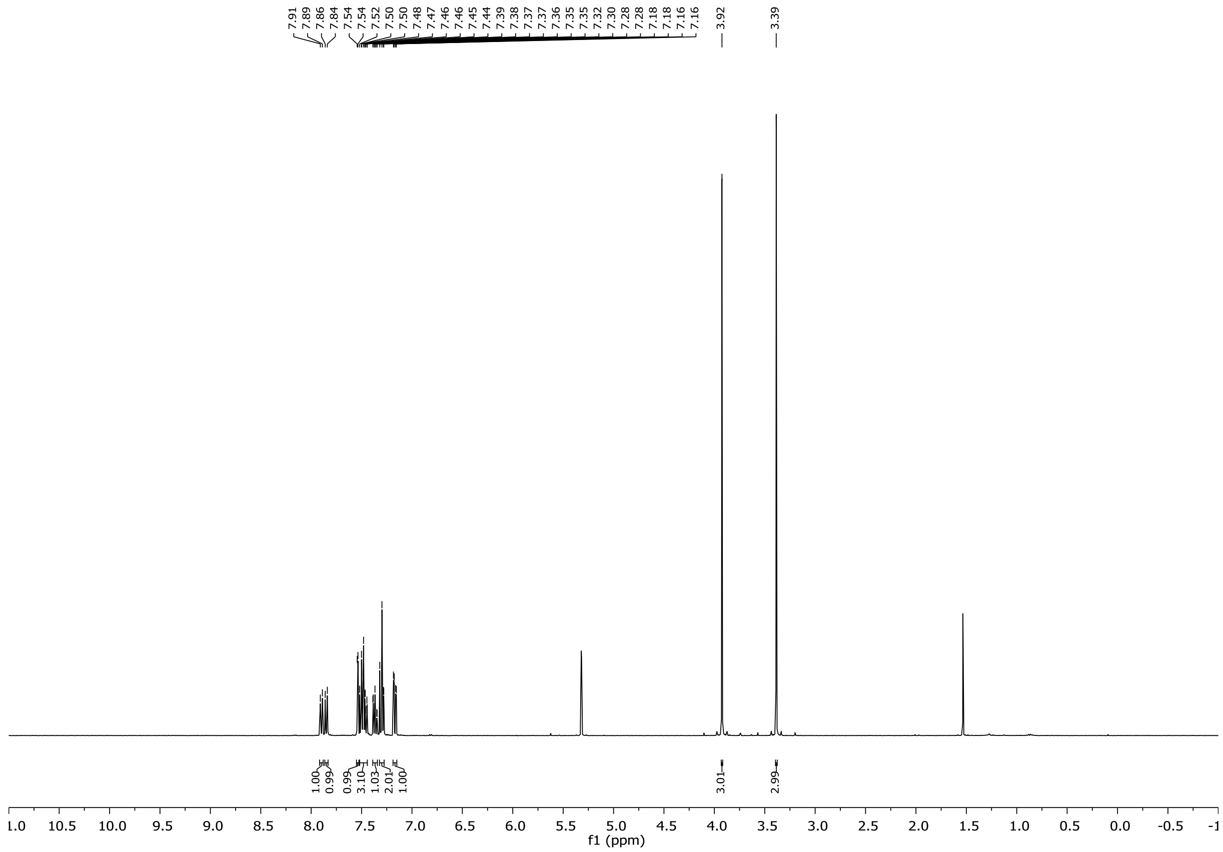

^{13}C NMR spectrum of methyl 5-methoxy-2-(naphthalen-1-yl)benzoate (15)

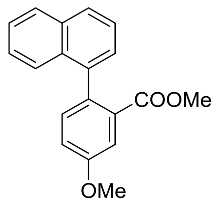

Frequency: 101 MHz

Solvent: CD_2Cl_2

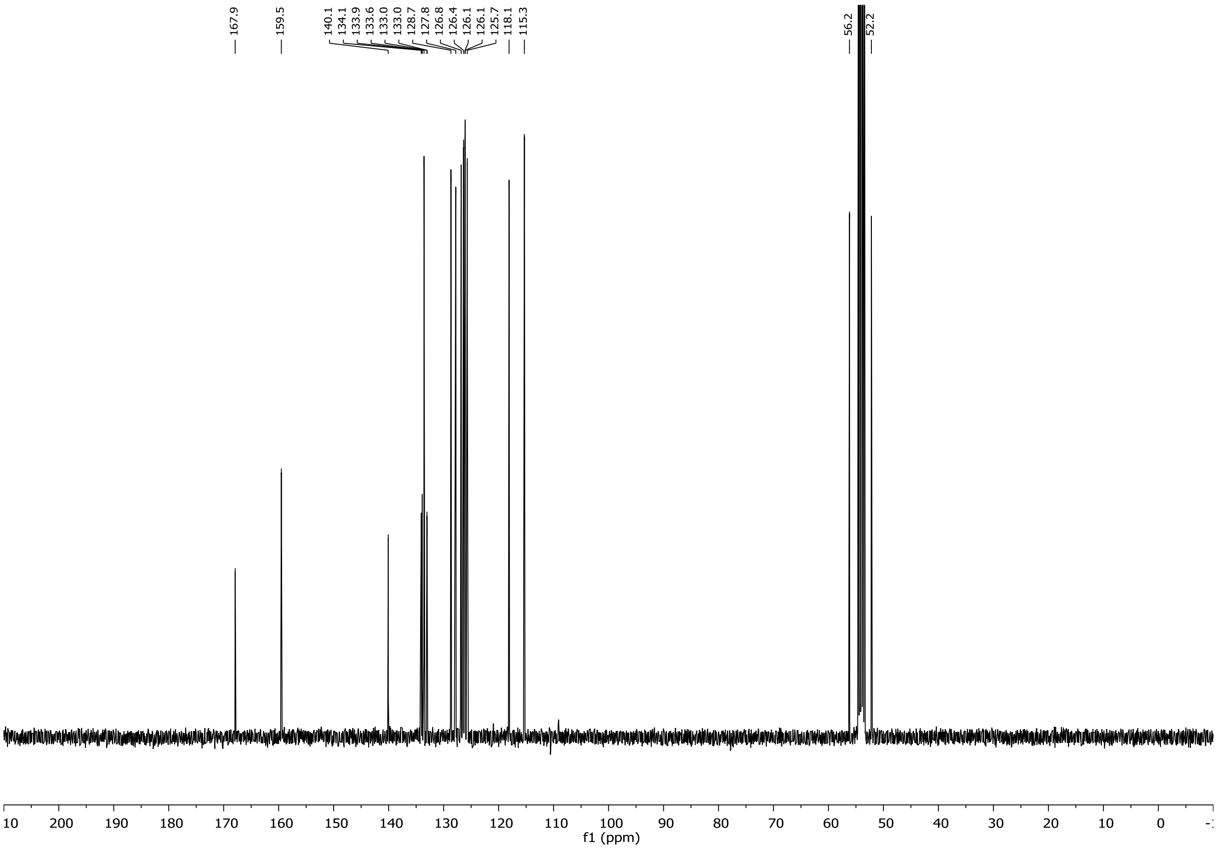

^{1}H NMR spectrum of *N*,*N*-diethyl-5-methoxy-2-(naphthalen-1-yl-)benzamide (16)

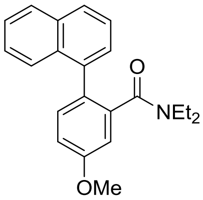

Frequency: 400 MHz

Solvent: $(CD_3)_2SO$

Temperature: 333 K

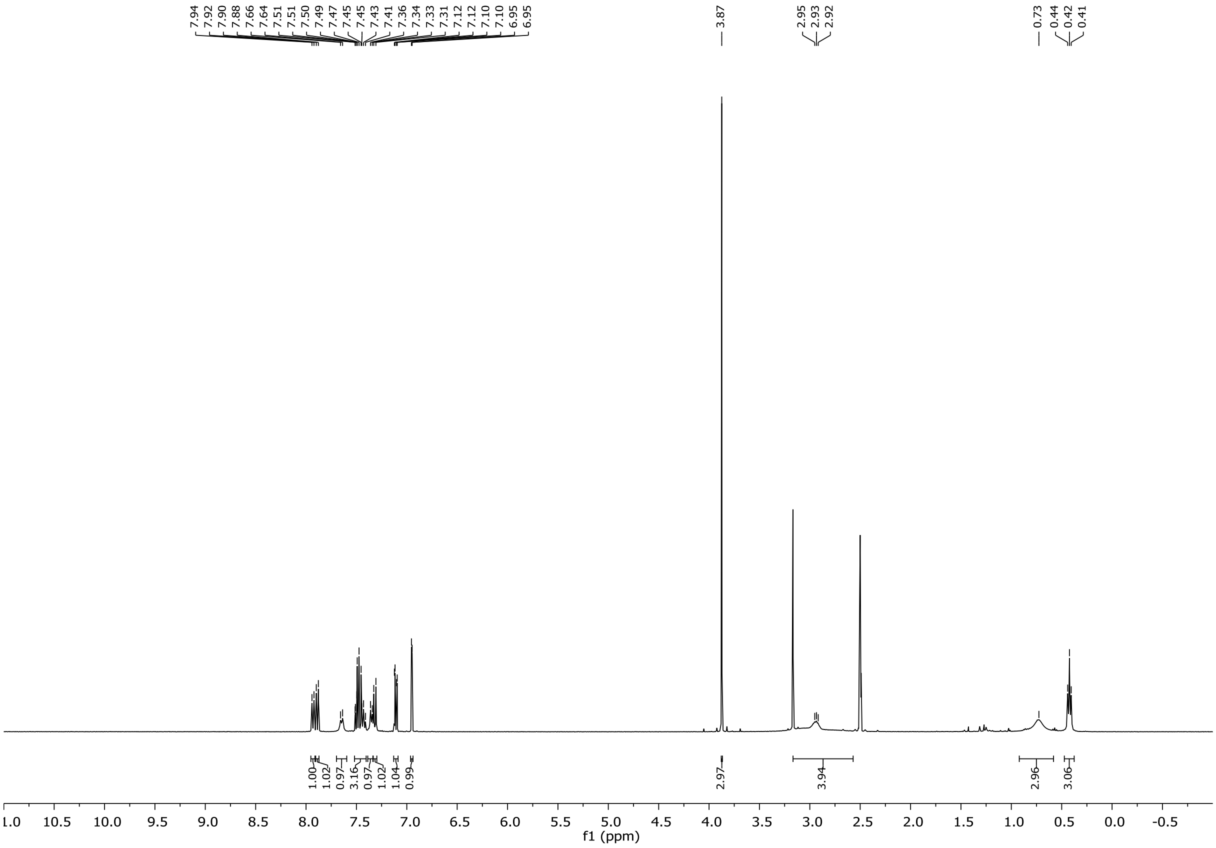

^{13}C NMR spectrum of *N*,*N*-diethyl-5-methoxy-2-(naphthalen-1-yl)benzamide (16)

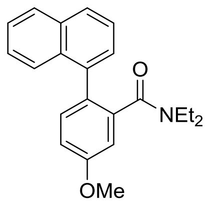

Frequency: 101 MHz

Solvent: $(CD_3)_2SO$

Temperature: 333 K

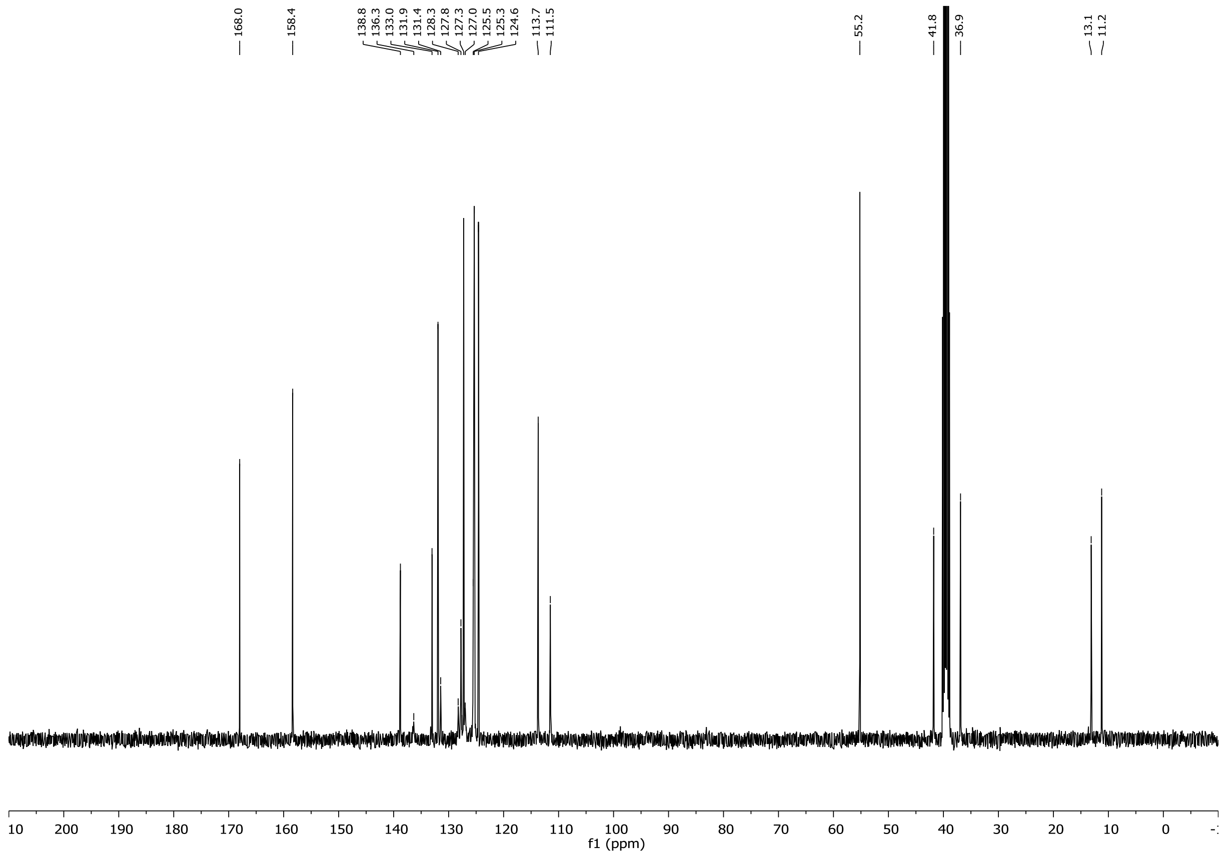

^{1}H NMR spectrum of 9-methoxy-7*H*-benzo[*c*]fluoren-7-one (17)

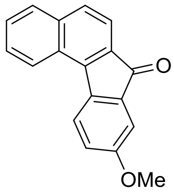

Frequency: 500 MHz

Solvent: CD_2Cl_2

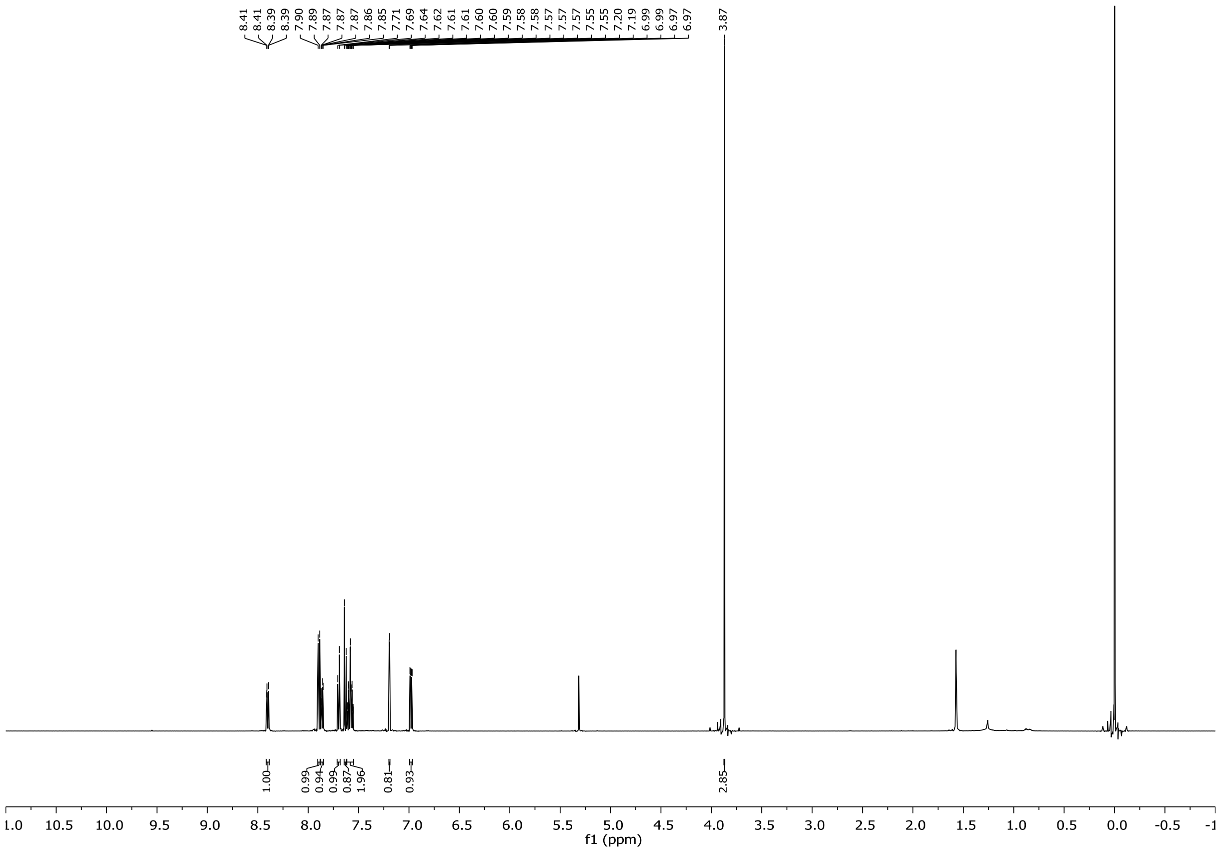

^{13}C NMR spectrum of 9-methoxy-7*H*-benzo[*c*]fluoren-7-one (17)

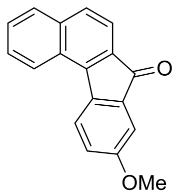

Frequency: 126 MHz

Solvent: CD_2Cl_2

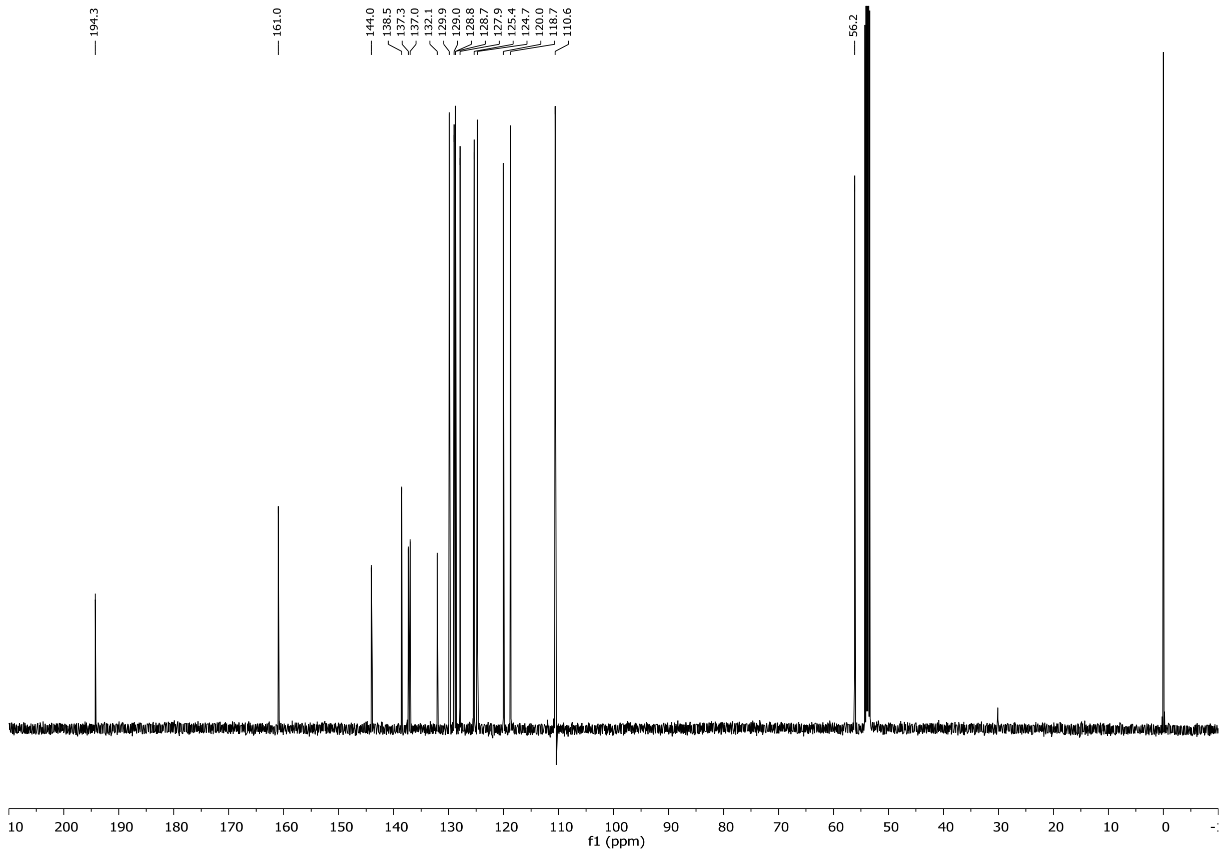

Comparison of ^{1}H NMR spectra of *N*,*N*-diethyl-5-methoxy-2-(6-methoxyisoquinolin-1-yl)benzamide (12) and partly deuterated 12-D

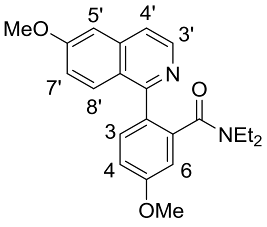

(**12**, top)

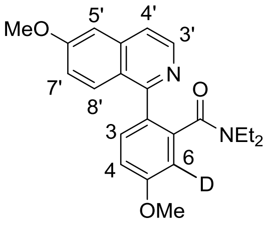

(**12-D**, bottom; about 40% deuterium incorporation)

Frequency: 500 MHz

Solvent: CD_2Cl_2

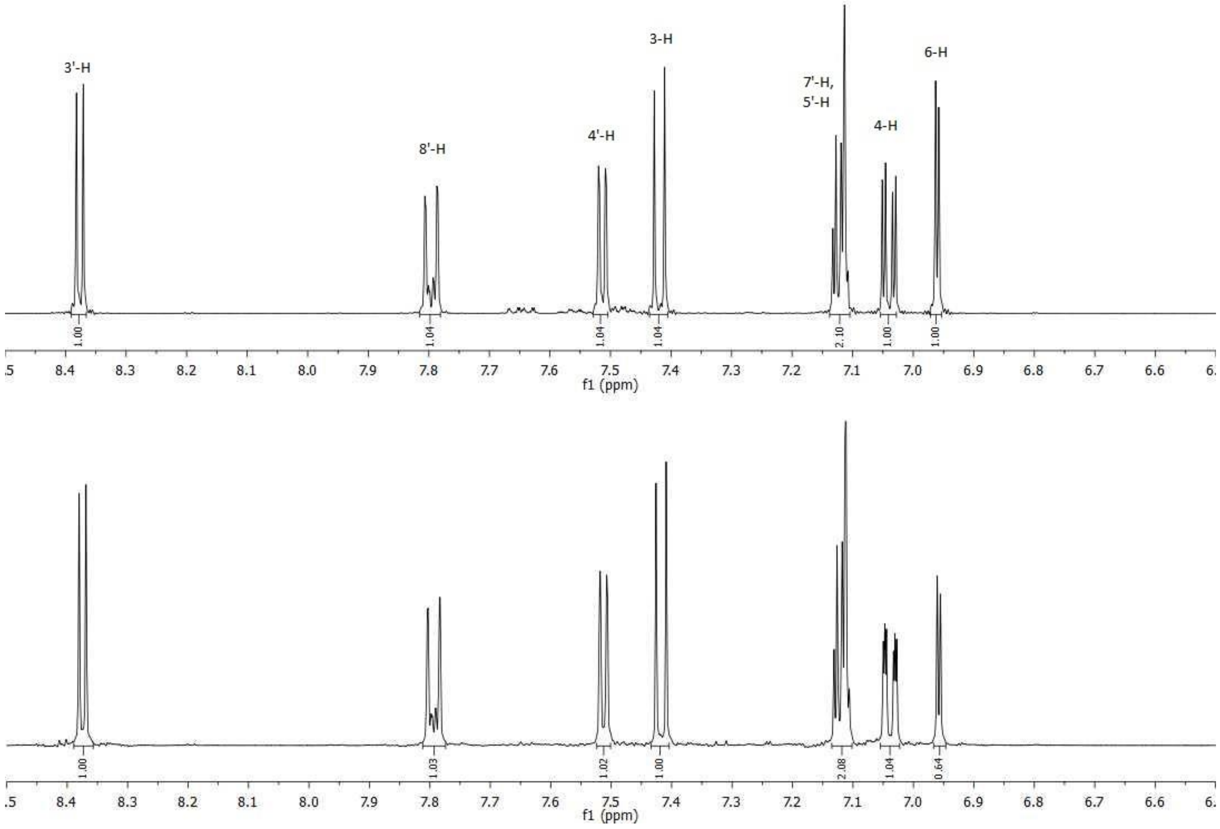

Comparison of ^{1}H NMR spectra of *N*,*N*-diethyl-5-methoxy-2-(napthalen-1-yl)benzamide (16) and partly deuterated 16-D

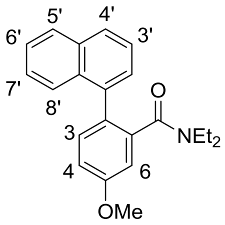

(**16**, top)

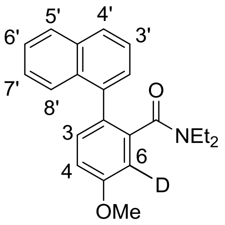

(**16-D**, bottom; about 20% deuterium incorporation)

Frequency: 400 MHz

Solvent: $(CD_3)_2SO$

Temperature: 333 K

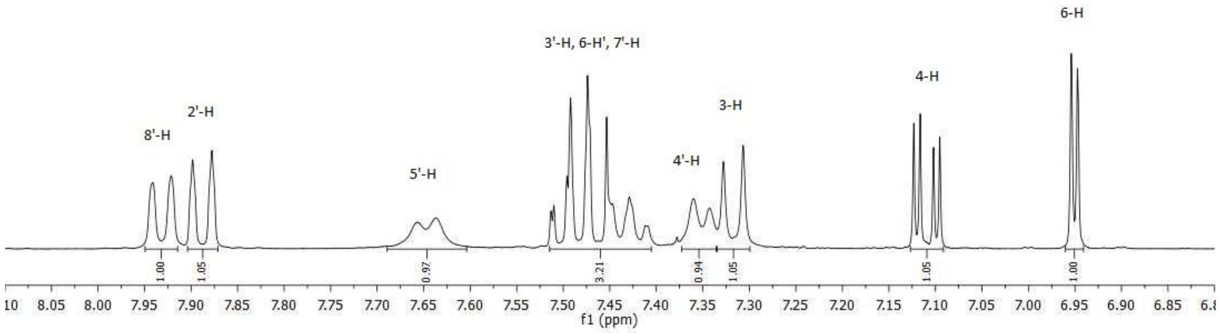

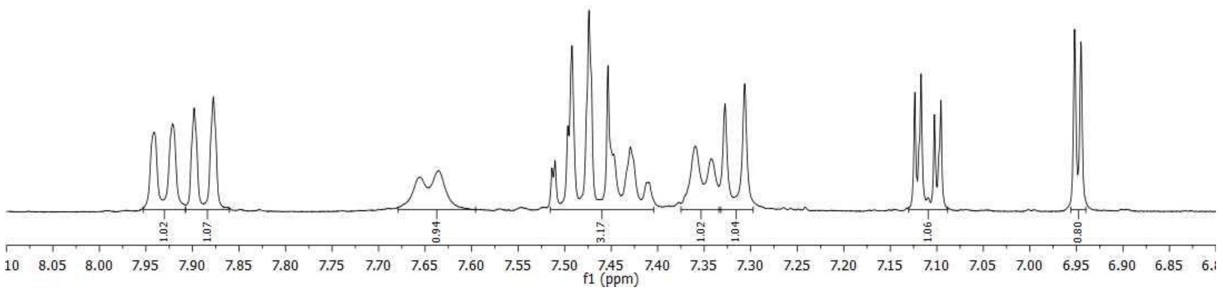

References

1. Boger, D. L.; Brotherton, C. E.; Kelley, M. D. *Tetrahedron* **1981**, *37*, 3977–3980.

2. Kucznierz, R.; Dickhaut, J.; Leinert, H.; von der Saal, W. *Synth. Commun.* **1999**, *29*, 1617–1625.

3. Krätzschmar, F.; Kaßel, M.; Delony, D.; Breder, A. *Chem. Eur. J.* **2015**, *21*, 7030–7037.

4. Metzger, A.; Schade, M. A.; Knochel, P. *Org. Lett.* **2008**, *10*, 1107–1110.

5. Kunitomo, J.; Satoh, M. *Tetrahedron* **1983**, *39*, 3261–3265.

6. Chaudhary, S.; Pecic, S.; LeGendre, O.; Harding, W. W. *Tetrahedron Lett.* **2009**, *50*, 2437–2439.

7. Kunitomo, J.; Miyata, Y. *Heterocycles* **1986**, *24*, 437–440.
